# Supplementary material for: Effectiveness of Psychological Treatments for Borderline Personality Disorder and Predictors of Treatment Outcomes: A Multivariate Multilevel Meta-Analysis of Data from All Design Types
Source: J Clin Med. 2021 Nov 29;10(23):5622. doi: 10.3390/jcm10235622 (PMC8658126; doi:10.3390/jcm10235622)
Supplement: Supplementary file 1 [file jcm-10-05622-s001.zip › jcm-1408872-Supplementary.pdf]

Supplementary Material S1: Prisma Checklist

Section/topic	#	Checklist item	Reported on page #
**TITLE**			
Title	1	Identify the report as a systematic review, meta-analysis, or both.	1
**ABSTRACT**			
Structured summary	2	Provide a structured summary including, as applicable: background; objectives; data sources; study eligibility criteria, participants, and interventions; study appraisal and synthesis methods; results; limitations; conclusions and implications of key findings; systematic review registration number.	1
**INTRODUCTION**			
Rationale	3	Describe the rationale for the review in the context of what is already known.	2-3
Objectives	4	Provide an explicit statement of questions being addressed with reference to participants, interventions, comparisons, outcomes, and study design (PICOS).	3
**METHODS**			
Protocol and registration	5	Indicate if a review protocol exists, if and where it can be accessed (e.g., Web address), and, if available, provide registration information including registration number.	3
Eligibility criteria	6	Specify study characteristics (e.g., PICOS, length of follow-up) and report characteristics (e.g., years considered, language, publication status) used as criteria for eligibility, giving rationale.	3-5
Information sources	7	Describe all information sources (e.g., databases with dates of coverage, contact with study authors to identify additional studies) in the search and date last searched.	3
Search	8	Present full electronic search strategy for at least one database, including any limits used, such that it could be repeated.	3 – Supplementary Material S2
Study selection	9	State the process for selecting studies (i.e., screening, eligibility, included in systematic review, and, if applicable, included in the meta-analysis).	3
Data collection process	10	Describe method of data extraction from reports (e.g., piloted forms, independently, in duplicate) and any processes for obtaining and confirming data from investigators.	3-5
Data items	11	List and define all variables for which data were sought (e.g., PICOS, funding sources) and any assumptions and	4-6

		simplifications made.	
Risk of bias in individual studies	12	Describe methods used for assessing risk of bias of individual studies (including specification of whether this was done at the study or outcome level), and how this information is to be used in any data synthesis.	6
Summary measures	13	State the principal summary measures (e.g., risk ratio, difference in means).	5-6
Synthesis of results	14	Describe the methods of handling data and combining results of studies, if done, including measures of consistency (e.g., I^2) for each meta-analysis.	5-6
Section/topic	#	Checklist item	Reported on page #
Risk of bias across studies	15	Specify any assessment of risk of bias that may affect the cumulative evidence (e.g., publication bias, selective reporting within studies).	6
Additional analyses	16	Describe methods of additional analyses (e.g., sensitivity or subgroup analyses, meta-regression), if done, indicating which were pre-specified.	6 & 7
**RESULTS**			
Study selection	17	Give numbers of studies screened, assessed for eligibility, and included in the review, with reasons for exclusions at each stage, ideally with a flow diagram.	7
Study characteristics	18	For each study, present characteristics for which data were extracted (e.g., study size, PICOS, follow-up period) and provide the citations.	Supplementary Material S6, 7
Risk of bias within studies	19	Present data on risk of bias of each study and, if available, any outcome level assessment (see item 12).	Dataset published online
Results of individual studies	20	For all outcomes considered (benefits or harms), present, for each study: (a) simple summary data for each intervention group (b) effect estimates and confidence intervals, ideally with a forest plot.	9-12 - Dataset published online
Synthesis of results	21	Present results of each meta-analysis done, including confidence intervals and measures of consistency.	7-14
Risk of bias across studies	22	Present results of any assessment of risk of bias across studies (see Item 15).	13-14 & Supplementary Materials
Additional analysis	23	Give results of additional analyses, if done (e.g., sensitivity or subgroup analyses, meta-regression [see Item 16]).	14
**DISCUSSION**			

Summary of evidence	24	Summarize the main findings including the strength of evidence for each main outcome; consider their relevance to key groups (e.g., healthcare providers, users, and policy makers).	14-16
Limitations	25	Discuss limitations at study and outcome level (e.g., risk of bias), and at review-level (e.g., incomplete retrieval of identified research, reporting bias).	17-18
Conclusions	26	Provide a general interpretation of the results in the context of other evidence, and implications for future research.	17-18
**FUNDING**			
Funding	27	Describe sources of funding for the systematic review and other support (e.g., supply of data); role of funders for the systematic review.	18

Supplementary Material S2

Search Strings and Results from the Most Recent Literature Update (March 2019 to October 2019)

Psychinfo (OVID)

1. (borderline personality disorder/ or borderline states/ or borderline personality).mp. (13495)
2. Limit 1 to yr= "2019-Current". (335)
3. (clinical trials/ or (clinical trial* or controlled trial* or RCT*).mp. (76403)
4. Limit 3 to yr= "2019-Current". (2999)
5. 2 and 4. (20)

Medline (OVID)

1. (borderline personality disorder/ or borderline personality).mp. (8734)
2. Limit 1 to yr= "2019-Current". (483)
3. (clinical trial/ or (clinical trial* or controlled trial* or RCT*).mp. (1372224)
4. Limit 3 to yr= "2019-Current". (52694)
5. 2 and 4. (34)

Embase (OVID)

1. (Borderline state/ OR Borderline personality).mp. (14162)
2. Limit 1 to yr= "2019-Current". (629)
3. (clinical trial/ OR (clinical trial* or controlled trial* or RCT*).mp. (2015129)
4. Limit 3 to yr= "2015-Current". (101600)
5. 2 and 4. (54)

Cochrane Library

1. ("borderline personality"):ti,ab,kw AND ("clinical trial*" OR "controlled trial*" OR RCT*):pt,ab,kw,ti. (97)

Supplementary Material S3:

Quality Rating Checklist

1. BPD diagnosis made according to semi-structured diagnostic interviews, such as the SCID (0 = no or unknown, 1 = yes, but with inadequate or unknown inter-rater reliability [IRR], 2 = yes, with adequate IRR);
2. If a treatment manual was used (0 = no or unknown, 1 = yes, but treatment manual is unpublished, 2 = yes, with published treatment manual);
3. If therapists were trained either specifically for the study or in a general training (0 = no or unknown, 1 = no or unknown, but therapists are clearly experts, 2 = yes);
4. If treatment integrity was checked (0 = no or unknown, 1 = yes, by supervision, 2 = yes by independent raters);
5. If data were analyzed using intent-to-treat (ITT) analyses (0 = no or unknown, 1 = yes, but ITT analysis is partly violated, 2 = yes);
6. If the study was randomized (0 = no or unknown, 1 = yes, but randomization is partly violated, 2 = yes);
7. If applicable, if randomization was independent and adequately concealed;
8. If assessment interviews were conducted by independent or blind assessors (0 = no or unknown, 1 = yes, independent or blind, 2 = yes, blind and independent);
9. If dropout was reported (0 = no, 1 = yes, but no distinction between types of dropout, 2 = yes, with adequate distinction between types of dropout)

Supplementary Material S4:

Estimation of Cohen's d from Binary Outcomes

In case of binary outcomes, Cohens' d was defined as:

$$d = \ln(\mathrm{OR})*\sqrt{(3)}/\pi \qquad \text{Eq. (S1)}$$

with ln(OR) (OR = odds-ratio) defined by:

$$\ln(\mathrm{OR}) = \ln(\mathrm{Odds}_{pre}) - \ln(\mathrm{Odds}_{post}) = \ln(p'_{pre}/(1-p'_{pre})) - \ln(p'_{post}/(1-p'_{post})) \qquad \text{Eq. (S2)}$$

and p'_{pre} = corrected proportion meeting the outcome at pretest, p'_{post} = corrected proportion meeting the outcome at posttest.

Corrected outcome proportions were calculated with the Agresti-Coull method:

$$p' = (n+2)/(N+4) \qquad \text{Eq. (S3)}$$

with n = observed number meeting the outcome and N = total number (Agresti & Coul, 1998).

Supplementary Material S5

Estimation of Hedges' g from Cohen's d

Cohen's d was transformed into Hedges' g by the following formula:

$$g = (1-3/(4*\mathrm{d.f.} - 1)) * d, \qquad \text{Eq. (S4)}$$

with d.f. = n-1, and n = the sample size

The variance of Hedges' g was defined by:

$$\sigma^2(g) = (\mathrm{d.f.}/ (n * (\mathrm{d.f.}-2))) + d\text{^}2 * (\mathrm{d.f.}-(\mathrm{d.f.}-2)/J\text{^}2)/ (\mathrm{d.f.}-2)) * J\text{^}2, \qquad \text{Eq. (S5)}$$

with J = (1-3/(4*d.f.-1))

For the transformed effect sizes, we used the logarithmic relationship between time in weeks and effect size to calculate the effect size at 1 year. Thus, the transformed Hedges' g was defined as follows:

$$Transformed.g = a * g / (\mathrm{LN}(\mathrm{time} +1) \qquad \text{Eq. (S6)}$$

With a = LN(53) and time = Duration in Weeks.

The standard error (SE), and thus the variance (by squaring the SE), of the transformed g was calculated with the same formula as transformed g, by replacing g by the SE.

Supplementary Material S6

Study Characteristics of Included Studies

Table S1. Characteristics and Demographics of all Included Studies

Author(s), year	Treatment	Country	N	Quality	Setting	Format	Trial Type	Mean Age	Mean Axis I	Mean Axis II	Prop. Males	Data analysis	Substance exclusion	ISCED level	Treatment Duration
Amianto, 2011	TAU	Italy	17	1.11	Outpatient	Individual	RCT	40.1	0.63	-	0.47	ITTunclear	Dependence	3	52 weeks
	PDT		18	1.11	Outpatient	Individual		39.2	0.63	-	0.56			3	48 weeks

Bales, 2012	MBT	Netherlands	45	1.43	Day trmt.	Combined	OT	30.1	1.90	0.62	0.30	ITTmodern	None	3	78 weeks
Barnicot, 2019	DBT<br>MBT	UK	58<br>32	0.89<br>0.89	Outpatient<br>Outpatient	Combined<br>Combined	CT	29.3<br>34.3	2.172<br>1.563	-<br>-	0.28<br>0.28	ITTmodern	None	-<br>-	52 weeks<br>52 weeks
Bateman, 1999	MBT<br>TAU	UK	22<br>19	1.33<br>1.00	Day trmt.<br>Outpatient	Combined<br>Individual	RCT	30.3<br>33.3	2.67<br>2.47	-<br>-	0.32<br>0.53	Completers	Abuse	3<br>2	78 weeks<br>78 weeks
Bateman, 2009	MBT<br>SCM	UK	71<br>63	1.56<br>1.38	Outpatient<br>Outpatient	Combined<br>Combined	RCT	31.3<br>30.9	3.03<br>3.08	1.39<br>1.23	0.20<br>0.22	ITTmodern	Cl. Detox	4<br>4	78 weeks<br>78 weeks
Bellino, 2010	IPT	Italy	27	1.11	Outpatient	Combined	RCT	26.2	-	-	0.30	Completers	Abuse	3	32 weeks
Ben-Porath, 2004	DBT	US	26	1.00	Day trmt.	Combined	OT	35.5	1.02	-	0.04	Completers	Abuse	3	26 weeks
Blum, 2008	TAU	US	59	0.80	Outpatient	Individual	RCT	31.6	-	2.7	0.14	ITTmodern	Abuse	3	20 weeks
Bohus, 2000	DBT	Germany	24	0.38	Inpatient	Combined	PS	28.3	-	-	0.00	ITTmodern	Dependence	-	12 weeks
Bohus, 2004	DBT<br>TAU	Germany	40<br>20	0.88<br>0.50	Inpatient<br>Inpatient	Combined<br>Combined	CT	29.1<br>29.5	1.46<br>1.81	-<br>-	0.00<br>0.00	Completers	Dependence	-<br>-	12 weeks<br>12 weeks
Borschman, 2013	TAU	UK	42	0.90	Outpatient	Individual	RCT	36.1	-	-	0.17	ITTmodern	None	-	26 weeks
Bos, 2010	TAU	Netherlands	37	0.60	Outpatient	Individual	RCT	31.8	-	-	0.11	Completers	None	-	18 weeks
Brassington, 2006	DBT	NZ	10	1.25	Outpatient	Combined	PS	34.3	-	-	0.00	ITTmodern	None	-	26 weeks
Brown, 2004	DBT	US	32	1.50	Outpatient	Individual	OT	29.0	2.71	0.72	0.12	ITT lofc	Cl. Detox	3	52 weeks
Carter, 2010	DBT<br>TAU+WL	Australia	38<br>35	1.33<br>0.89	Outpatient<br>Outpatient	Combined<br>Individual	RCT	24.5<br>24.7	2.30<br>2.30	-<br>-	0.00<br>0.00	Completers	Cl. Detox	3<br>3	24 weeks<br>24 weeks
**Author(s), year**	**Treatment**	**Country**	**N**	**Quality**	**Setting**	**Format**	**Trial Type**	**Mean Age**	**Mean Axis I**	**Mean Axis II**	**Prop. Males**	**Data analysis**	**Substance exclusion**	**ISCED level**	**Treatment duration**

Clarkin, 2001	TFP	US	23	1.25	Outpatient	Individual	OT	32.7	1.65	2.94	0.00	ITT lofc	None	-	52 weeks
Clarkin, 2007	TFP	US	30	1.44	Outpatient	Individual	RCT	30.9	2.33	-	0.08	Completers	Dependence	4	52 weeks
	DBT		30	1.44	Outpatient	Combined		30.9	2.33	-	0.08			4	52 weeks
	PDT		30	1.44	Outpatient	Individual		30.9	2.33	-	0.08			4	52 weeks
Comtois, 2007	DBT	US	38	0.88	Outpatient	Combined	OT	34.0	2.55	-	0.04	Completers	Dependence	-	52 weeks
Cottraux, 2009	CBT	France	31	1.56	Outpatient	Individual	RCT	34.3	3.09	1.12	0.28	Completers	Dependence	5	52 weeks
	CCT		31	1.56	Outpatient	Individual		32.6	3.09	1.27	0.18			5	52 weeks
Davidson, 2006	DBT+TAU	UK	54	1.89	Outpatient	Individual	RCT	32.4	-	-	0.17	ITT lofc	Dependence	3	52 weeks
	TAU		52	1.22	Outpatient	Individual		31.4	-	-	0.15			2	52 weeks
Del Pozo, 2017	Mixed	Germany	269	0.86	Inpatient	Combined	OT	35.0	3.00	0.39	0.16	ITTmodern	Dependence	3	13 weeks
Dickhaut, 2014	ST	Netherlands	18	1.29	Outpatient	Combined	PS	28.5	2.38	0.89	0.00	ITTmodern	Cl. Detox	3	104 weeks
Doering, 2010	TFP	Austria	52	2.00	Outpatient	Individual	RCT	27.5	1.60	-	0.00	ITT lofc	Dependence	-	52 weeks
	CTBE		52	1.67	Outpatient	Individual		27.2	1.50	-	0.00			-	52 weeks
Farrell, 2009	ST	US	16	1.11	Outpatient	Combined	RCT	35.3	-	-	0.00	Completers	None	3	30 weeks
	TAU		16	0.67	Outpatient	Individual		35.9	-	-	0.00			3	30 weeks
Fassbinder, 2016	ST	Germany	10	1.25	Outpatient	Group	PS	35.0	5.1	1.80	0.00	ITTmodern	Cl. Detox	-	52 weeks
Feigenbaum, 2012	DBT	UK	25	1.56	Outpatient	Combined	RCT	35.4	2.00	1.20	0.28	ITTmodern	Cl. Detox	2	52 weeks
	TAU		16	1.00	Outpatient	Individual		34.6	2.17	1.10	0.25			3	52 weeks
Friedrich, 2003	DBT	Germany	33	0.43	Outpatient	Combined	Nat.	33.4	3.18	0.12	0.09	Completers	None	4	52 weeks
Giessen-Bloo, 2006	ST	Netherlands	44	1.56	Outpatient	Individual	RCT	31.7	2.95	2.14	0.09	ITT lofc	Cl. Detox	3	156 weeks
	TFP		42	1.56	Outpatient	Individual		29.5	2.40	2.05	0.05			3	156 weeks

Gratz, 2006	Mixed	US	36	0.63	Day trmt.	Combined	PS	28.6	-	-	0.22	Completers	Dependence	3	12 weeks
Gratz & Gunderson, 2006	TAU	US	14	1.25	Outpatient	Combined	PS	33.3	-	-	0.00	Completers	Dependence	4	14 weeks
Harley, 2007	DBTmin + TAU	US	49	0.71	Outpatient	Combined	Nat.	39.1	2.05	-	0.08	Completers	None	4	30 weeks

**Author(s), year**	**Treatment**	**Country**	**N**	**Quality**	**Setting**	**Format**	**Trial Type**	**Mean Age**	**Mean Axis I**	**Mean Axis II**	**Prop. Males**	**Data analysis**	**Substance exclusion**	**ISCED level**	**Treatment duration**
Höschel, 2006	DBT	Germany	26	0.29	Inpatient	Combined	Nat.	28.3	-	-	0.13	Completers	Dependence	-	12 weeks
Jacob, 2018	ST	Germany	13	0.50	Outpatient	Individual	Nat	28.4	4.08	1.08	0.15	ITT lofc	Cl.Detox	2	52 weeks
Jorgensen, 2012	MBT<br>PDT	Denmark	58<br>27	1.44<br>1.22	Outpatient<br>Outpatient	Combined<br>Group	RCT	29.5<br>29.7	1.77<br>1.44	0.65<br>0.65	0.03<br>0.07	Completers	Cl. Detox	3<br>3	104 weeks<br>104 weeks
Kellet, 2013	CAT	US	19	1.13	Outpatient	Individual	OT	30.0	-	-	0.17	Completers	None	-	26 weeks
Koons, 2001	DBT<br>TAU	US	14<br>14	1.56<br>1.00	Outpatient<br>Outpatient	Combined<br>Individual	RCT	34.5<br>35.4	-<br>-	-<br>-	0.00<br>0.00	Completers	Dependence	-<br>-	26 weeks<br>26 weeks
Korner, 2006	PDT<br>TAU	Australia	29<br>31	0.88<br>0.25	Outpatient<br>Outpatient	Individual<br>Individual	Nat.	27.9<br>29.7	-<br>-	-<br>-	0.48<br>0.41	Completers	Cl. Detox	-<br>-	52 weeks<br>52 weeks
Kröger, 2006	DBT	Germany	50	0.88	Inpatient	Combined	OT	30.5	5.50	-	0.12	ITT lofc	Cl. Detox	-	13 weeks
Kröger, 2013	DBT	Germany	1423	1.25	Inpatient	Combined	OT	32.0	3.70	0.90	0.25	ITTmodern	Cl. Detox	3	12 weeks
Kröger, 2015	PDT	Germany	269	1.43	Inpatient	Combined	OT	35.0	3.01	0.26	0.16	ITTmodern	Cl. Detox	3	12 weeks
Laurenssen, 2018	MBT<br>TAU	Netherlands	54<br>41	1.33<br>1.11	Day trmt.<br>Outpatient	Combined<br>Combined	RCT	34.0<br>34.0	2.33<br>1.61	0.70<br>0.54	0.22<br>0.18	ITTmodern	Cl. Detox	-<br>-	78 weeks<br>78 weeks
Leerer, 1997	DBTmin	US	14	0.88	Inpatient	Group	OT	31.9	-	-	-	Completers	Cl. Detox	3	52 weeks

Leppänen, 2016	Mixed<br>TAU	Finland	24<br>47	1.00<br>0.78	Outpatient<br>Outpatient	Combined<br>Individual	RCT	31.9<br>32.3	-<br>-	-<br>-	0.16<br>0.13	Completers	Abuse	-<br>-	52 weeks<br>52 weeks
Linehan, 1991	DBT<br>TAU	US	13<br>13	1.67<br>1.11	Outpatient<br>Outpatient	Combined<br>Individual	RCT	26.7<br>26.7	-<br>-	-<br>-	0.00<br>0.00	ITT lofc	Dependence	4<br>4	52 weeks<br>52 weeks
Linehan, 2006	DBT<br>CTBE	US	52<br>49	1.89<br>1.56	Outpatient<br>Outpatient	Combined<br>Individual	RCT	29.0<br>29.6	2.81<br>3.20	0.38<br>0.41	0.00<br>0.00	ITTmodern	None	4<br>4	52 weeks<br>52 weeks
Linehan, 2008	DBT	US	12	1.67	Outpatient	Combined	PS	36.8	2.50	1.17	0.00	ITTmodern	Dependence	3	26 weeks

**Author(s), year**	**Treatment**	**Country**	**N**	**Quality**	**Setting**	**Format**	**Trial Type**	**Mean Age**	**Mean Axis I**	**Mean Axis II**	**Prop. Males**	**Data analysis**	**Substance exclusion**	**ISCED level**	**Treatment duration**
Linehan, 2015	DBT<br>DBTmin + TAU<br>DBTmin + TAU	US	33<br>33<br>33	1.89<br>1.89<br>1.89	Outpatient<br>Outpatient<br>Outpatient	Combined<br>Combined<br>Combined	RCT	31.2<br>30.1<br>29.8	2.12<br>2.06<br>1.97	0.97<br>0.76<br>0.49	0.00<br>0.00<br>0.00	ITTmodern	None	3<br>3<br>3	52 weeks<br>52 weeks<br>52 weeks
Löf, 2018	MBT	Sweden	75	1.29	Outpatient	Combined	Nat.	30.4	3.10	1.21	0.11	ITTmodern	Cl. Detox	5	78 weeks
Löffler-Stastka 2006	PDT	Austria	20	0.57	Inpatient	Combined	PS	38.2	-	-	0.50	Completers	Cl. Detox	3	6 weeks
Lyng, 2019	DBTyouth<br>DBT	Ireland	19<br>11	0.63<br>0.63	Outpatient<br>Outpatient	Combined<br>Combined	CT	20.5<br>21.5	0.83<br>1.08	-	0.17<br>0.31	Completers	Dependence	3<br>3	52 weeks<br>52 weeks
Markowitz, 2007	IPT	US	8	1.00	Outpatient	Group	PS	34.0	2.05	0-.92	0.13	Completers	None	-	34 weeks

McMain, 2009	DBT<br>GPM	Canada	90<br>90	2.00<br>1.89	Outpatient<br>Outpatient	Combined<br>Individual	RCT	29.4<br>31.3	2.12<br>2.03	0.67<br>0.66	0.10<br>0.18	ITTmodern	Dependence	3<br>3	52 weeks<br>52 weeks
McMain, 2017	DBTmin<br>TAU	Canada	42<br>42	1.67<br>1.11	Outpatient<br>Outpatient	Group<br>Individual	RCT	27.3<br>32.1	2.14<br>2.36	-<br>-	0.17<br>0.26	ITTmodern	None	4<br>4	20 weeks<br>20 weeks
Meares, 1999	PDT	Australia	48	1.00	Outpatient	Individual	CT	29.4	-	-	0.37	Completers	Cl. Detox	3	52 weeks
Moen, 2012	DBT	US	5	1.67	Outpatient	Combined	RCT	37.0	-	-	0.40	ITT lofc	Dependence	-	16 weeks
Morey, 2010	CBT	US	8	1.33	Outpatient	Individual	PS	29.6	-	-	0.25	ITT lofc	None	3	6 weeks
Nadort, 2009	ST<br>ST	Netherlands	32<br>30	1.89<br>1.89	Outpatient<br>Outpatient	Individual<br>Individual	RCT	31.8<br>32.1	2.16<br>2.40	-<br>-	0.03<br>0.03	ITT lofc	Cl. Detox	3<br>3	78 weeks<br>78 weeks
Nordahl, 2005	ST	Norway	6	0.71	Outpatient	Individual	CS	25.6	1.83	0.67	0.00	ITTmodern	None	-	95 weeks
Nordahl, 2019	MCT	Norway	12	1.14	Outpatient	Individual	OT	23.1	4.70	-	0.17	ITTmodern	Dependence	-	52 weeks
Nysaeter, 2010	PDT	Norway	32	0.88	Outpatient	Individual	Nat.	28.9	2.82	1.74	0.19	ITT lofc	Cl. Detox	3	156 weeks
Pascienzy, 2011	DBT<br>TAU + WL	Australia	43<br>47	0.88<br>0.25	Outpatient<br>Outpatient	Combined<br>Individual	CT	33.6<br>33.6	2.42<br>2.33	-<br>-	0.05<br>0.08	Completers	None	-<br>-	26 weeks<br>26 weeks
Perez, 2016	TFP	US	10	0.63	Outpatient	Individual	OT	27.8	1.00	0.60	0.00	ITTmodern	None	4	52 weeks
Perry, 2017	PDT	Canada	15	0.25	Outpatient	Individual	OT	26.8	3.44	-	0.13	ITTmodern	Abuse	-	156 weeks

**Author(s), year**	**Treatment**	**Country**	**N**	**Quality**	**Setting**	**Format**	**Trial Type**	**Mean Age**	**Mean Axis I**	**Mean Axis II**	**Prop. Males**	**Data analysis**	**Substance exclusion**	**ISCED level**	**Treatment duration**
Prendergast, 2007	DBT	Australia	16	0.88	Outpatient	Combined	OT	36.4	1.18	-	0.00	Completers	None	-	26 weeks
Priebe, 2012	DBT<br>TAU	UK	40<br>40	1.44<br>1.00	Outpatient<br>Outpatient	Combined<br>Combined	RCT	33.0<br>31.3	7.50<br>8.50	3.40<br>3.50	0.13<br>0.13	Completers	None	-<br>-	52 weeks<br>52 weeks

Reiss, 2014-1	ST	US	42	1.14	Inpatient	Combined	PS	36.4	-	-	0.03	Completers	None	3	18 weeks
Reiss, 2014-2	ST	US	37	1.14	Inpatient	Combined	PS	31.6	-	-	0.11	Completers	None	3	12 weeks
Reiss, 2014-3	ST	Germany	15	1.00	Inpatient	Combined	PS	25.5	-	-	0.00	Completers	None	3	10 weeks
Rizvi, 2017	DBT	US	50	1.13	Outpatient	Combined	OT	29.5	2.84	0.88	0.20	ITTmodern	None	4	26 weeks
Ryle, 2000	CAT	UK	27	1.00	Outpatient	Individual	CS	34.3	0.98	-	0.41	Completers	None	3	24 weeks
Sachdeva, 2013	DBT	US	25	1.13	Outpatient	Combined	CT	36.6	4.44	-	0.16	ITTlofc	None	-	52 weeks
	DDP		27	1.00	Outpatient	Individual		28.0	4.28	-	0.15			-	52 weeks
	TAU		16	0.33	Outpatient	Combined		29.3	5.13	-	0.31			-	52 weeks
Sachse, 2011	CBT	UK	22	1.00	Outpatient	Group	PS	39.0	1.86	0.27	0.14	ITTunclear	Cl. Detox	3	8 weeks
Simpson, 2004	DBT	US	13	1.33	Outpatient	Combined	RCT	32.7	0.91	-	0.00	Completers	Dependence	3	12 weeks
Sinnaeve, 2018	DBT	Netherlands	42	1.56	Outpatient	Combined	RCT	25.6	1.35	-	0.05	Completers	Cl. Detox	-	52 weeks
	DBT		42	1.56	Inpatient	Combined		26.2	1.35	-	0.05			-	39 weeks
Smits, 2019	MBT	Netherlands	44	1.50	Outpatient	Combined	RCT	29.9	2.60	1.50	0.20	ITTmodern	None	3	78 weeks
	MBT		47	1.50	Day trmt.	Combined		31.4	2.60	1.30	0.16			3	78 weeks
Soler, 2005	DBTmin	Spain	39	1.00	Outpatient	Group	RCT	26.3	-	-	0.10	ITT lofc	Dependence	-	12 weeks
Soler, 2009	DBTmin	Spain	29	1.44	Outpatient	Group	RCT	28.5	-	-	0.21	ITTmodern	Dependence	-	13 weeks
	TAU		30	1.00	Outpatient	Group		30.0	-	-	0.13			-	13 weeks
Sollberger, 2014	TAU	Switserland	21	0.43	Inpatient	Combined	OT	29.4	2.42	0.50	0.25	Completers	Abuse	-	12 weeks
	Mixed		34	1.00	Inpatient	Combined		26.7	2.42	0.88	0.19			-	12 weeks
Stanley, 2007	DBT	US	20	0.75	Outpatient	Combined	PS	32.2	-	-	0.15	Completers	None	-	26 weeks
Stiglmayer, 2014	DBT	Germany	70	1.50	Outpatient	Combined	Nat.	30.1	2.04	0.68	0.09	Completers	Dependence	3	52 weeks

Turner, 2000	DBTmin<br>CCT	US	12<br>12	1.67<br>1.67	Outpatient<br>Outpatient	Combined<br>Combined	RCT	22.0<br>22.0	2.54<br>2.54	1.21<br>1.21	0.21<br>0.21	ITTunclear	Cl. Detox	3<br>3	52 weeks<br>52 weeks
**Author(s), year**	**Treatment**	**Country**	**N**	**Quality**	**Setting**	**Format**	**Trial Type**	**Mean Age**	**Mean Axis I**	**Mean Axis II**	**Prop. Males**	**Data analysis**	**Substance exclusion**	**ISCED level**	**Treatment duration**
Vaillancourt, 2017	DBT	US	29	0.75	Outpatient	Combined	Nat.	35.9	2.48	0.14	0.04	Completers	Dependence	5	59 weeks
Van den Bosch, 2013	DBT	Netherlands	39	1.13	Day trmt.	Combined	PS	26.0	-	-	0.00	ITT lofc	Cl. Detox	3	14 weeks
Verheul, 2003	DBT<br>TAU	Netherlands	27<br>31	1.44<br>0.89	Outpatient<br>Outpatient	Combined<br>Individual	RCT	35.1<br>34.7	-<br>-	-<br>-	0.00<br>0.00	ITTmodern	None	3<br>4	52 weeks<br>52 weeks
Weinberg, 2006	TAU	US	15	0.80	Outpatient	Combined	RCT	26.3	-	-	0.00	ITTmodern	Dependence	4	14 weeks
Wilberg, 1998	Th. Com<br>Th. Com	Norway	31<br>12	0.50<br>0.50	Day trmt.<br>Day trmt.	Combined<br>Combined	Nat.	32.0<br>27.0	1.10<br>0.75	-<br>-	0.29<br>0.08	Completers	None	-<br>-	82 weeks<br>104 weeks
Wildgoose, 2001	CAT	UK	5	1.14	Outpatient	Group	PS	39.4	-	-	0.40	Completers	Abuse	-	16 weeks

Notes. WL = Wait list, US = United States of America, UK = United Kingdom, Day trmt = Day treatment, OT = Open Trial, PS = Pilot Study, Nat = Naturalistic study, CS = Case series, CT = Controlled trial, Cl. Detox = Exclusion of Clinical Detox.

Supplementary Material S7
References of Included Studies

1. Amianto, F., Ferrero, A., Pierò, A., Cairo, E., Rocca, G., Simonelli, B., Fassina, S., Abbate-Daga, G. & Fassino, S. (2011). Supervised team management, with or without structured psychotherapy, in heavy users of a mental health service with borderline personality disorder: a two-year follow-up preliminary randomized study. *BMC Psychiatry*, *11*(1), 181.
2. Bales, D., van Beek, N., Smits, M., Willemsen, S., Busschbach, J. J., Verheul, R., & Andrea, H. (2012). Treatment outcome of 18-month, day hospital mentalization-based treatment (MBT) in patients with severe borderline personality disorder in the Netherlands. *Journal of Personality Disorders*, *26*(4), 568-582.
3. Barnicot, K., & Crawford, M. (2019). Dialectical behaviour therapy v. mentalisation-based therapy for borderline personality disorder. *Psychological Medicine*, *49*(12), 2060-2068.

4. Bateman, A., & Fonagy, P. (1999). Effectiveness of partial hospitalization in the treatment of borderline personality disorder: a randomized controlled trial. *American Journal of Psychiatry*, *156*(10), 1563-1569.
5. Bateman, A., & Fonagy, P. (2001). Treatment of borderline personality disorder with psychoanalytically oriented partial hospitalization: an 18-month follow-up. *American Journal of Psychiatry*, *158*(1), 36-42.
6. Bateman, A., & Fonagy, P. (2008). 8-year follow-up of patients treated for borderline personality disorder: mentalization-based treatment versus treatment a usual. *American Journal of Psychiatry*, *165*(5), 631-638. [see also Bateman & Fonagy, 1999]
7. Bateman, A., & Fonagy, P. (2009). Randomized controlled trial of outpatient mentalization-based treatment versus structured clinical management for borderline personality disorder. *The American Journal of Psychiatry*, *166*(12), 1355-1364.
8. Bellino, S., Rinaldi, C., & Bogetto, F. (2010). Adaptation of interpersonal psychotherapy to borderline personality disorder: a comparison of combined therapy and single pharmacotherapy. *Canadian Journal of Psychiatry*, *55*(2), 74-81. [see also Bozzatello, 2016]
9. Ben-Porath, D. D., Peterson, G. A., & Smee, J. (2004). Treatment of individuals with borderline personality disorder using dialectical behavior therapy in a community mental health setting: Clinical application and a preliminary investigation. *Cognitive and Behavioral Practice*, *11*(4), 424-434.
10. Blum, N., John, D. S., Pfohl, B., Stuart, S., Mccormick, B., Allen, F., Arndt, S., & Black, D. W. (2008). Systems Training for Emotional Predictability and Problem Solving (STEPPS) for Outpatients With Borderline Personality Disorder : A Randomized Controlled Trial and 1-Year Follow-Up. *The American Journal of Psychiatry, 165*, 468–478.
11. Bohus, M., Haaf, B., Simms, T., Limberger, M. F., Schmahl, C., Unckel, C., Lieb, K. & Linehan, M. M. (2004). Effectiveness of inpatient dialectical behavioral therapy for borderline personality disorder: a controlled trial. *Behaviour Research and Therapy*, *42*(5), 487-499. [see also Kleindienst et al., 2011]
12. Bohus, M., Haaf, B., Stiglmayr, C., Pohl, U., Böhme, R., & Linehan, M. (2000). Evaluation of inpatient dialectical-behavioral therapy for borderline personality disorder—a prospective study. *Behaviour Research and Therapy*, *38*(9), 875-887.
13. Borschmann, R., Barrett, B., Hellier, J. M., Byford, S., Henderson, C., Rose, D., … Moran, P. (2013). Joint crisis plans for people with borderline personality disorder: feasibility and outcomes in a randomised controlled trial. *The British Journal of Psychiatry : The Journal of Mental Science*, *202*(5), 357–364.
14. Bos, E. H., van Wel, E. B., Appelo, M. T., & Verbraak, M. J. P. M. (2010). A randomized controlled trial of a Dutch version of systems training for emotional predictability and problem solving for borderline personality disorder. *The Journal of Nervous and Mental Disease*, *198*(4), 299–304.
15. Brassington, J., & Krawitz, R. (2006). Australasian dialectical behaviour therapy pilot outcome study: effectiveness, utility and feasibility. *Australian Psychiatry*, *14*(3), 313-318.

16. Brown, G. K., Newman, C. F., Charlesworth, S. E., Crits-Christoph, P., & Beck, A. T. (2004). An open clinical trial of cognitive therapy for borderline personality disorder. *Journal of Personality Disorders*, *18*, 257-271.
17. Carter, G. L., Willcox, C. H., Lewin, T. J., Conrad, A. M., & Bendit, N. (2010). Hunter DBT project: randomized controlled trial of dialectical behaviour therapy in women with borderline personality disorder. *Australian and New Zealand Journal of Psychiatry*, *44*(2), 162-173.
18. Clarkin, J. F., Foelsch, P. A., Levy, K. N., Hull, J. W., Delaney, J. C., & Kernberg, O. F. (2001). The development of a psychodynamic treatment for patients with borderline personality disorder: a preliminary study of behavioral change. *Journal of Personality Disorders*, *15*(6), 487-495. [see also Clarkin et al., 2005]
19. Clarkin, J. F., Levy, K. N., & Schiavi, J. M. (2005). Transference focused psychotherapy: development of a psychodynamic treatment for severe personality disorders. *Clinical Neuroscience Research*, *4*(5), 379-386. [see also Clarkin et al., 2001]
20. Clarkin, J. F., Levy, K. N., Lenzenweger, M. F., & Kernberg, O. F. (2007). Evaluating three treatments for borderline personality disorder: a multiwave study. *American Journal of Psychiatry*, *164*(6), 922-928.
21. Comtois, K. A., Elwood, L., Holdcraft, L. C., Smith, W. R., & Simpson, T. L. (2007). Effectiveness of dialectical behavior therapy in a community mental health center. *Cognitive and Behavioral Practice*, *14*(4), 406-414.
22. Cottraux, J., Boutitie, F., Milliery, M., Genouihlac, V., Yao, S. N., Mollard, E., Bonasse, F., Gaillard, S., Djamoussain, D., de Mey Guillard, C., Culem, A. & Gueyffier, F. (2009). Cognitive therapy versus Rogerian supportive therapy in borderline personality disorder. *Psychotherapy and Psychosomatics*, *78*(5), 307-316.
23. Coyle, T. N., Shaver, J. A., & Linehan, M. M. (2018). On the potential for iatrogenic effects of psychiatric crisis services: The example of dialectical behavior therapy for adult women with borderline personality disorder. *Journal of Consulting and Clinical Psychology*, *86*(2), 116-124. [see also Linehan, 2006]
24. Davidson, K., Norrie, J., Tyrer, P., Gumley, A., Tata, P., Murray, H., & Palmer, S. (2006). The effectiveness of cognitive behavior therapy for borderline personality disorder: results from the borderline personality disorder study of cognitive therapy (BOSCOT) trial. *Journal of Personality Disorders*, *20*(5), 450-465. [see also Davidson et al., 2010]
25. Davidson, K. M., Tyrer, P., Norrie, J., Palmer, S. J., & Tyrer, H. (2010). Cognitive therapy v. usual treatment for borderline personality disorder: prospective 6-year follow-up. *The British Journal of Psychiatry*, *197*(6), 456–462. [see also Davidson et al., 2006]
26. Del Pozo, M., Kliem, S., Mestel, R., Votsmeier-Röhr, A., & Kröger, C. (2018). Psychodynamic oriented Multimodal therapy for inpatients with borderline personality disorder: effectiveness, response, and dropout. *Psychotherapie, Psychosomatik, Medizinische Psychologie*, *68*(2), 82-90.
27. Dickhaut, V., & Arntz, A. (2014). Combined group and individual schema therapy for borderline personality disorder: a pilot study. *Journal of Behavior Therapy and Experimental Psychiatry*, *45*(2), 242-251.

28. Doering, S., Hörz, S., Rentrop, M., Fischer-Kern, M., Schuster, P., Benecke, C., Buchheim, A., Marius, P. & Buchheim, P. (2010). Transference-focused psychotherapy v. treatment by community psychotherapists for borderline personality disorder: randomised controlled trial. *The British Journal of Psychiatry*, *196*(5), 389-395.
29. Euler, S., Stalujanis, E., Allenbach, G., Kolly, S., De Roten, Y., Despland, J. N., & Kramer, U. (2018). Dialectical behavior therapy skills training affects defense mechanisms in borderline personality disorder: an integrative approach of mechanisms in psychotherapy. *Psychotherapy Research*, 1-12. [see also Kramer et al., 2016]
30. Farrell, J. M., Shaw, I. A., & Webber, M. A. (2009). A schema-focused approach to group psychotherapy for outpatients with borderline personality disorder: a randomized controlled trial. *Journal of Behavior Therapy and Experimental Psychiatry*, *40*(2), 317-328.
31. Fassbinder, E., Rudolf, S., Bussiek, A., Kröger, C., Arnold, R., Greggersen, W., Hüppe. M., Sipos, V. & Schweiger, U. (2007). Effektivität der dialektischen Verhaltenstherapie bei Patienten mit Borderline-Persönlichkeitsstörung im Langzeitverlauf–Eine 30-Monats-Katamnese nach stationärer Behandlung. Psychotherapie, Psychosomatik, *Medizinische Psychologie, 57*, 161-169. [see also Kröger et al., 2006]
32. Fassbinder, E., Schuetze, M., Kranich, A., Sipos, V., Hohagen, F., Shaw, I., … & Schweiger, U. (2016). Feasibility of group schema therapy for outpatients with severe borderline personality disorder in Germany: A pilot study with three year follow-up. *Frontiers in Psychology*, *7*, 1851.
33. Feigenbaum, J. D., Fonagy, P., Pilling, S., Jones, A., Wildgoose, A., & Bebbington, P. E. (2012). A real-world study of the effectiveness of DBT in the UK National Health Service. *British Journal of Clinical Psychology*, *51*(2), 121-141.
34. Fischer-Kern, M., Doering, S., Taubner, S., Hörz, S., Zimmermann, J., Rentrop, M., … & Buchheim, A. (2015). Transference-focused psychotherapy for borderline personality disorder: Change in reflective function. *The British Journal of Psychiatry*, *207*(2), 173-174.
35. Friedrich, J., Gunia, H., & Huppertz, M. (2003). Evaluation eines ambulanten Netzwerks für Dialektisch Behaviorale Therapie. *Verhaltenstherapie & Verhaltensmedizin*, *24*, 289-306
36. Giesen-Bloo, J., Van Dyck, R., Spinhoven, P., Van Tilburg, W., Dirksen, C., Van Asselt, T., Kremers, I., Nadort, N. & Arntz, A. (2006). Outpatient psychotherapy for borderline personality disorder: randomized trial of schema-focused therapy vs transference-focused psychotherapy. *Archives of General Psychiatry*, *63*(6), 649-658.
37. Gratz, K. L., & Gunderson, J. G. (2006). Preliminary data on an acceptance-based emotion regulation group intervention for deliberate self-harm among women with borderline personality disorder. *Behavior Therapy*, *37*(1), 25-35.
38. Gratz, K. L., Lacroce, D. M., & Gunderson, J. G. (2006). Measuring changes in symptoms relevant to borderline personality disorder following short-term treatment across partial hospital and intensive outpatient levels of care. *Journal of Psychiatric Practice*, *12*(3), 153-159.
39. Gregory, R. J., & Sachdeva, S. (2016). Naturalistic Outcomes of Evidence-Based Therapies for Borderline Personality Disorder at a Medical University Clinic. *The American Journal of Psychotherapy, 70*(2), 167–185. [see also Sachdeva et al., 2013]

40. Harley, R. M., Baity, M. R., Blais, M. A., & Jacobo, M. C. (2007). Use of dialectical behavior therapy skills training for borderline personality disorder in a naturalistic setting. *Psychotherapy Research*, *17*(3), 351-358.
41. Harned, M. S., Chapman, A. L., Dexter-Mazza, E. T., Murray, A., Comtois, K. a., & Linehan, M. M. (2009). Treating co-occurring Axis I disorders in recurrently suicidal women with borderline personality disorder: A 2-year randomized trial of dialectical behavior therapy versus community treatment by experts. *Personality Disorders: Theory, Research, and Treatment*, (1), 35–45. [see also Linehan et al., 2006]
42. Höschel, K. (2006). Dialektisch-Behaviorale Therapie der Borderline-Persönlichkeitsstörung in der Regelversorgung–das Saarbrücker Modell. *Verhaltenstherapie*, *16*(1), 17–24.
43. Jacob, G. A., Hauer, A., Köhne, S., Assmann, N., Schaich, A., Schweiger, U., & Fassbinder, E. (2018). A Schema Therapy–Based eHealth Program for Patients with Borderline Personality Disorder (priovi): Naturalistic Single-Arm Observational Study. *JMIR Mental Health*, *5*(4), e10983.
44. Jørgensen, C. R., Freund, C., Bøye, R., Jordet, H., Andersen, D., & Kjølbye, M. (2013). Outcome of mentalization-based and supportive psychotherapy in patients with borderline personality disorder: a randomized trial. *Acta Psychiatrica Scandinavica*, 127(4), 305-317. [see also Jørgensen et al., 2014]
45. Jørgensen, C. R., Bøye, R., Andersen, D., Døssing Blaabjerg, A. H., Freund, C., Jordet, H., & Kjølbye, M. (2014). Eighteen months post-treatment naturalistic follow-up study of mentalization-based therapy and supportive group treatment of borderline personality disorder: Clinical outcomes and functioning. *Nordic Psychology*, *66*(4), 254–273. [see also Jørgensen et al., 2013]
46. Kellett, S., Bennett, D., Ryle, T., & Thake, A. (2013). Cognitive Analytic Therapy for Borderline Personality Disorder : Therapist Competence and Therapeutic Effectiveness in Routine Practice, *Clinical Psychology and Psychotherapy, 225*, 216–225.
47. Kleindienst, N., Limberger, M. F., Ebner-Priemer, U. W., Keibel-Mauchnik, J., Dyer, A., Berger, M.…Bohus, M. (2011). Dissociation predicts poor response to dialectical behavioral therapy in female patients with borderline personality disorder. *Journal ofPersonality Disorders, 25(*4), 432-447. [see also Bohus et al., 2004]
48. Koons, C. R., Robins, C. J., Tweed, J. L., Lynch, T. R., Gonzalez, A. M., Morse, J. Q., … & Bastian, L. A. (2001). Efficacy of dialectical behavior therapy in women veterans with borderline personality disorder. *Behavior Therapy*, *32*(2), 371-390.
49. Korner, A., Gerull, F., Meares, R., & Stevenson, J. (2006). Borderline personality disorder treated with the conversational model. *Comprehensive Psychiatry, 47*, 406– 411. [see also Korner et al., 2008]
50. Korner, A., Hons, M., Psychothei, M. M., Meares, R., & D, M. (2008). The Nothing that is Something: Core Dysphoria as the Central Feature of Borderline Personality DisorderImplications for Treatment. *The American Journal of Psychotherapy, 62(4)*, 377– 395. [see also Korner et al., 2006]
51. Kröger, C., Schweiger, U., Sipos, V., Arnold, R., Kahl, K. G., Schunert, T., Rudolf, S. & Reinecker, H. (2006). Effectiveness of dialectical behaviour therapy for borderline personality disorder in an inpatient

setting. *Behaviour Research and Therapy*, *44*(8), 1211-1217. [see also Fassbinder et al., 2007]

52. Kröger, C., Harbeck, S., Armbrust, M., & Kliem, S. (2013). Effectiveness, response, and dropout of dialectical behavior therapy for borderline personality disorder in an inpatient setting. *Behaviour Research and Therapy, 51*(8), 411-416.
53. Kröger, C. Del Pozo, M. Mestel, R. Votsmeijer-Röhr, A. & Kliem, S. (2015). Effectiveness, response, and dropout in psychodynamic therapy for inpatients with borderline personality disorder. An open trial in routine mental healthcare. *Submitted for publication.*
54. Laurenssen, E. M., Luyten, P., Kikkert, M. J., Westra, D., Peen, J., Soons, M. B., … & Dekker, J. J. (2018). Day hospital mentalization-based treatment v. specialist treatment as usual in patients with borderline personality disorder: randomized controlled trial. *Psychological Medicine*, *48*(15), 2522-2529.
55. Leerer, C. G. (1997). Outcomes of inpatient cognitive-behavioral treatment of borderline personality disorder. *Dissertation Abstracts International B: Science and, Engineering*.
56. Leppänen, V., Hakko, H., Sintonen, H., & Lindeman, S. (2016). Comparing effectiveness of treatments for borderline personality disorder in communal mental health care: the Oulu BPD Study. *Community Mental Health Journal*, *52*(2), 216-227. [see also Leppänen et al., 2015]
57. Leppänen, V., Kärki, A., Saariaho, T., Lindeman, S., & Hakko, H. (2015). Changes in schemas of patients with severe borderline personality disorder: The Oulu BPD study. *Scandinavian Journal of Psychology*, *56*(1), 78-85. [see also Leppänen et al., 2016]
58. Linehan, M. M., Armstrong, H. E., Suarez, A., Allmon, D., & Heard, H. L. (1991). Cognitive-behavioral treatment of chronically parasuicidal borderline patients. *Archives of General Psychiatry*, *48*(12), 1060-1064. [see also Linehan et al., 1993; Linehan et al, 1994]
59. Linehan, M.M., Heard, H.L., & Armstrong, H.E. (1993). Naturalistic Follow-up of a Behavioral Treatment for Chronically Parasuicidal Borderline Patients. *Archives of General Psychiatry, 50, 971-974.* [see also Linehan et al., 1991]
60. Linehan, M. M., Tutek, D. A., Heard, H. L., & Armstrong, H. E. (1994). Interpersonal Outcome of Cognitive Behavioral Treatment for Chronically Suicidal Borderline Patients. *The American Journal of Psychiatry, 151, 12*, 1771. [see also Linehan et al., 1991]
61. Linehan, M. M., Comtois, K. A., Murray, A. M., Brown, M. Z., Gallop, R. J., Heard, H. L., Korslund, K.E., Tutek, D.A., Reynolds, S.K. & Lindenboim, N. (2006). Two-year randomized controlled trial and follow-up of dialectical behavior therapy vs therapy by experts for suicidal behaviors and borderline personality disorder. *Archives of General Psychiatry,63*(7), 757- 766. [see also Neacsiu et al., 2010; Coyle et al., 2018]
62. Linehan, M. M., McDavid, J. D., Brown, M. Z., Sayrs, J. H., & Gallop, R. J. (2008). Olanzapine plus dialectical behavior therapy for women with high irritability who meet criteria for borderline personality disorder: a double-blind, placebo-controlled pilot study. *Journal of Clinical Psychiatry*, *69*(6), 999-1005.
63. Linehan, M. M., Korslund, K. E., Harned, M. S., Gallop, R. J., Lungu, A., Neacsiu, A. D., McDavid, J., Comtois, K.A. & Murray-Gregory, A. M. (2015). Dialectical behavior therapy for high suicide risk in

individuals with borderline personality disorder: a randomized clinical trial and component analysis. *JAMA Psychiatry*, *72*(5), 475-482.

64. Löf, J., Clinton, D., Kaldo, V., & Rydén, G. (2018). Symptom, alexithymia and self-image outcomes of Mentalisation-based treatment for borderline personality disorder: a naturalistic study. *BMC Psychiatry*, *18*(1), 185.
65. Löffler-Stastka, H., Ponocny-Seliger, E., Meißel, T., & Springer-Kremser, M. (2006). Gender aspects in the planning of psychotherapy for borderline personality disorder. *Wiener Klinische Wochenschrift*, *118*(5-6), 160-169.
66. Lyng, J., Swales, M. A., Hastings, R. P., Millar, T., & Duffy, D. J. (2019). Outcomes for 18 to 25-year-olds with borderline personality disorder in a dedicated young adult only DBT programme compared to a general adult DBT programme for all ages 18+. *Early Intervention in Psychiatry*, *14,* 61-68.
67. Markowitz, J. C., Bleiberg, K., Pessin, H., & Skodol, A. E. (2007). Adapting interpersonal psychotherapy for borderline personality disorder. *Journal of Mental Health*, *16*(1), 103-116.
68. McMain, S. F., Links, P. S., Gnam, W. H., Guimond, T., Cardish, R. J., Korman, L., & Streiner, D. L. (2009). A randomized trial of dialectical behavior therapy versus general psychiatric management for borderline personality disorder. *The American Journal of Psychiatry*, *166*(12), 1365-1374. [see also McMain et al., 2012]
69. McMain, S. F., Guimond, T., Barnhart, R., Habinski, L., & Streiner, D. L. (2017). A randomized trial of brief dialectical behaviour therapy skills training in suicidal patients suffering from borderline disorder. *Acta Psychiatrica Scandinavica*, *135*(2), 138-148.
70. McMain SF, Guimond T, Streiner DL, Cardish RJ, Links PS (2012): Dialectical behavior therapy compared with general psychiatric management for borderline personality disorder: Clinical outcomes and functioning over a 2-year follow-up. *The American Journal of Psychiatry 169*, 650 – 661. [see also McMain et al., 2009]
71. Meares, R., Stevenson, J., & Comerford, A. (1999). Psychotherapy with borderline patients: I. A comparison between treated and untreated cohorts. *Australian and New Zealand Journal of Psychiatry*, *33*(4), 467-472. [see also Stevenson et al., 1992, 2005]
72. Moen, R., Freitag, M., Miller, M., Lee, S., Romine, A., Song, S., Adityanjee, A. & Schulz, S. C. (2012). Efficacy of extended-release divalproex combined with "condensed" dialectical behavior therapy for individuals with borderline personality disorder. *Annals of Clinical Psychiatry*, *24*(4), 255-60.
73. Morey, L. C., Lowmaster, S. E., & Hopwood, C. J. (2010). A pilot study of manual-assisted cognitive therapy with a therapeutic assessment augmentation for borderline personality disorder. *Psychiatry Research*, *178*(3), 531-535.
74. Nadort, M., Arntz, A., Smit, J. H., Giesen-Bloo, J., Eikelenboom, M., Spinhoven, P., van Asselt., Wensing, M. & van Dyck, R. (2009). Implementation of outpatient schema therapy for borderline personality disorder with versus without crisis support by the therapist outside office hours: A randomized trial. *Behaviour Research and Therapy*, *47*(11), 961-973.
75. Neacsiu, A. D., Lungu, A., Harned, M. S., Rizvi, S. L., & Linehan, M. M. (2014). Impact of dialectical behavior therapy versus community treatment by experts on emotional experience, expression, and

acceptance in borderline personality disorder. *Behaviour Research and Therapy*, *53*, 47–54. [see also Linehan et al., 2006]

76. Nordahl, H. M., & Nysæter, T. E. (2005). Schema therapy for patients with borderline personality disorder: a single case series. *Journal of Behavior Therapy and Experimental Psychiatry*, *36*(3), 254-264.
77. Nordahl, H. M., & Wells, A. (2019). Metacognitive Therapy of Early Traumatized Patients With Borderline Personality Disorder: A Phase-II Baseline Controlled Trial. *Frontiers in Psychology*, *10*, 1694.
78. Nysaeter, T. E., Nordahl, H. M., & Havik, O. E. (2010). A preliminary study of the naturalistic course of non-manualized psychotherapy for outpatients with borderline personality disorder: Patient characteristics, attrition and outcome. *Nordic Journal of Psychiatry*, *64*(2), 87–93.
79. Pasieczny, N., & Connor, J. (2011). The effectiveness of dialectical behaviour therapy in routine public mental health settings: an Australian controlled trial. *Behaviour Research and Therapy*, *49*(1), 4-10.
80. Perez, D. L., Vago, D. R., Pan, H., Root, J., Tuescher, O., Fuchs, B. H., & Lenzenweger, M. F. (2016). Frontolimbic neural circuit changes in emotional processing and inhibitory control associated with clinical improvement following transference-focused psychotherapy in borderline personality disorder. *Psychiatry and Clinical Neurosciences*, *70*(1), 51-61.
81. Perry, J. C., Bond, M., & Békés, V. (2017). The rate of improvement in long-term dynamic psychotherapy for borderline personality disorder. *The Journal of Nervous and Mental Disease*, *205*(7), 517-524.
82. Prendergast, N., & McCausland, J. (2007). Dialectic behaviour therapy: a 12-month collaborative program in a local community setting. *Behaviour Change*, *24*(01), 25- 35.
83. Priebe, S., Bhatti, N., Barnicot, K., Bremner, S., Gaglia, A., Katsakou, C., Molosankwe. I., McCrone, P. & Zinkler, M. (2012). Effectiveness and cost-effectiveness of dialectical behaviour therapy 15it hand-harming patients with personality disorder: a pragmatic randomised controlled trial. *Psychotherapy and Psychosomatics*,*81*(6), 356-365.
84. Reiss, N., Lieb, K., Arntz, A., Shaw, I. A., & Farrell, J. (2014). Responding to the treatment challenge of patients with severe BPD: results of three pilot studies of inpatient schema therapy. *Behavioural and Cognitive Psychotherapy*, *42(*03), 355-367.
85. Rizvi, S. L., Hughes, C. D., Hittman, A. D., & Vieira Oliveira, P. (2017). Can trainees effectively deliver dialectical behavior therapy for individuals with borderline personality disorder? Outcomes from a training clinic. *Journal of Clinical Psychology*, *73*(12), 1599-1611.
86. Ryle, A., & Golynkina, K. (2000). Effectiveness of time-limited cognitive analytic therapy of borderline personality disorder: Factors associated with outcome. *British Journal of Medical Psychology*, *73*(2), 197-210.
87. Sachdeva, S., Goldman, G., Mustata, G., Deranja, E., & Gregory, R. J. (2013). Naturalistic outcomes of evidence-based therapies for borderline personality disorder at a university clinic: a quasi-randomized trial. *Journal of the American Psychoanalytic Association*, *61(*3), 578–84. [see also Gregory & Sachdeva., 2016]
88. Sachse, S., Keville, S., & Feigenbaum, J. (2011). A feasibility study of mindfulness-based cognitive therapy for individuals with borderline

personality disorder. *Psychology and Psychotherapy: Theory, Research and Practice*, *84*(2), 184-200.

89. Simpson, E. B., Yen, S., Costello, E., Rosen, K., Begin, A., Pistorello, J., & Pearlstein, T. (2004).Combined dialectical behavior therapy and fluoxetine in the treatment of borderline personality disorder. *Journal of Clinical Psychiatry*, *65*, 3.
90. Sinnaeve, R., van den Bosch, L. M., Hakkaart-van Roijen, L., & Vansteelandt, K. (2018). Effectiveness of step-down versus outpatient dialectical behaviour therapy for patients with severe levels of borderline personality disorder: a pragmatic randomized controlled trial. *Borderline Personality Disorder and Emotion Dysregulation*, *5*(1), 12.
91. Smits, M. L., Feenstra, D. J., Eeren, H. V., Bales, D. L., Laurenssen, E. M., Blankers, M., … & Luyten, P. (2019). Day hospital versus intensive out-patient mentalisation-based treatment for borderline personality disorder: multicentre randomised clinical trial. *The British Journal of Psychiatry*, 1-6.
92. Soler, J., Pascual, J. C., Campins, J., Barrachina, J., Puigdemont, D., Alvarez, E., & Pérez, V. (2005). Double-blind, placebo-controlled study of dialectical behavior therapy plus olanzapine for borderline personality disorder. *American Journal of Psychiatry*. *162, 1221–1224.*
93. Soler, J., Pascual, J. C., Tiana, T., Cebrià, A., Barrachina, J., Campins, M. J., Gich, I., Alvarez. E. & Pérez, V. (2009). Dialectical behaviour therapy skills training compared to standard group therapy in borderline personality disorder: a 3-month randomised controlled clinical trial. *Behaviour Research and Therapy*, *47*(5), 353-358.
94. Sollberger, D., Gremaud-Heitz, D., Riemenschneider, A., Agarwalla, P., Benecke, C., Schwald, O., Küchenhoff, J., Walter, M. & Dammann, G. (2014). Change in identity diffusion and psychopathology in a specialized inpatient treatment for borderline personality disorder. *Clinical Psychology & Psychotherapy*, *22*(6), 559–69.
95. Stanley, B., Brodsky, B., Nelson, J. D., & Dulit, R. (2007). Brief dialectical behavior therapy (DBT-B) for suicidal behavior and non-suicidal self injury. *Archives of Suicide Research*, *11*(4), 337-341.
96. Stiglmayr, C., Stecher-mohr, J., Wagner, T., Mei, J., Spretz, D., Steffens, C., … Renneberg, B. (2014). Effectiveness of dialectic behavioral therapy in routine outpatient care : the Berlin Borderline Study. *Borderline Personality Disorder and Emotion Dysregulation*, 1-20.
97. Stevenson, J. & Meares, R. (1992). An outcome study of psychotherapy for patients with borderline personality disorder. *The American Journal of Psychiatry, 149*, 358–362. [see also Meares et al., 1999; Stevenson et al., 2005; Stevenson & Meares, 1999]
98. Stevenson, J., & Meares, R. (1999). Psychotherapy with borderline patients: II. A preliminary cost benefit study. *Australian & New Zealand Journal of Psychiatry*, *33*(4), 473-4770. [see also Meares et al., 1999; Stevenson et al., 2005; Stevenson & Meares, 1992]
99. Stevenson, J., Meares, R., & D'Angelo, R. (2005). Five-year outcome of outpatient psychotherapy with borderline patients. *Psychological Medicine, 35*, 79–87. [see also Meares et al., 1999; Stevenson & Meares, 1992]
100. Stiglmayr, C., Stecher-Mohr, J., Wagner, T., Meiβner, J., Spretz, D., Steffens, C., Roepke, S., Fydrich, T., Salbach-Andrae, H., Schulze, J. & Renneberg, B. (2014). Effectiveness of dialectic behavioral therapy in routine outpatient care: the Berlin Borderline Study. *Borderline Personality Disorder and Emotion Dysregulation*, *1*(1), 1-20.

101. Turner, R. M. (2000). Naturalistic evaluation of dialectical behavior therapy-oriented treatment for borderline personality disorder. *Cognitive and Behavioral Practice*, *7*(4), 413-419.
102. Vaillancourt, K. E. (2012). *Outpatient Dialectical Behavior Therapy at a Community Mental Health Center: Outcome Study* (Doctoral dissertation, Antioch University).
103. Van Asselt, A. D. I., Dirksen, C. D., Arntz, A., Giesen-Bloo, J. H., van Dyck, R., Spinhoven, P., … Severens, J. L. (2008). Out-patient psychotherapy for borderline personality disorder: cost-effectiveness of schema-focused therapy v. transference-focused psychotherapy. *The British Journal of Psychiatry: The Journal of Mental Science*, *192*(6), 450–457.
104. Van den Bosch, L. M. C., Koeter, M. W. J., Stijnen, T., Verheul, R., & van den Brink, W. (2005). Sustained efficacy of dialectical behaviour therapy for borderline personality disorder. *Behaviour Research and Therapy*, *43*(9), 1231–1241.
105. Van den Bosch, L.M.C., Sinnaeve, R. & Nijs, M. (2013). Kortdurende klinische dialectische gedragstherapie voor borderlinepersoonlijkheidsstoornis: Ontwerp van een programma en resultaten pilotstudie. *Tijdschrift voor Psychiatrie, 55*, 165-175.
106. Van den Bosch, L. M., Verheul, R., Schippers, G. M., & van den Brink, W. (2002). Dialectical behavior therapy of borderline patients with and without substance use problems: Implementation and long-term effects. *Addictive Behaviors*, *27*(6), 911-923.
107. Vaglum, P., Friis, S., Irion, T., Johns, S., Karterud, S., Larsen, F., & Vaglum, S. (1990). Treatment response of severe and nonsevere personality disorders in a therapeutic community day unit. *Journal of Personality Disorders*, *4*(2), 161-172.
108. Verheul, R., van den Bosch, L. M., Koeter, M. W., De Ridder, M. A., Stijnen, T., & Van Den Brink, W. (2003). Dialectical behaviour therapy for women with borderline personality disorder. *The British Journal of Psychiatry*, *182*(2), 135-140. [see also Van den Bosch 2002; 2005]
109. Wilberg, T., Friis, S., Karterud, S., Mehlum, L., Urnes, Ø., & Vaglum, P. (1998). Outpatient group psychotherapy: A valuable continuation treatment for patients with borderline personality disorder treated in a day hospital?: A 3-year follow-up study. *Nordic Journal of Psychiatry*, *52*(3), 213-221.
110. Wildgoose, A., Clarke, S., & Waller, G. (2001). Treating personality fragmentation and dissociation in borderline personality disorder: a pilot study of the impact of cognitive analytic therapy. *British Journal of Medical Psychology*, *74*(1), 47-55.

Supplementary Material S8

Funnel plots for the Final Models of the Transformed Effect Sizes

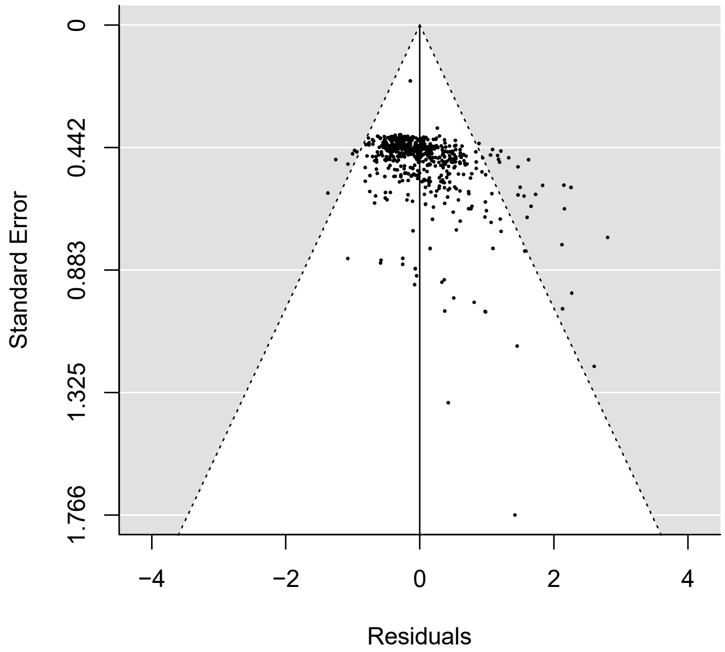

Figure S1. Funnel plot of the transformed effect sizes.

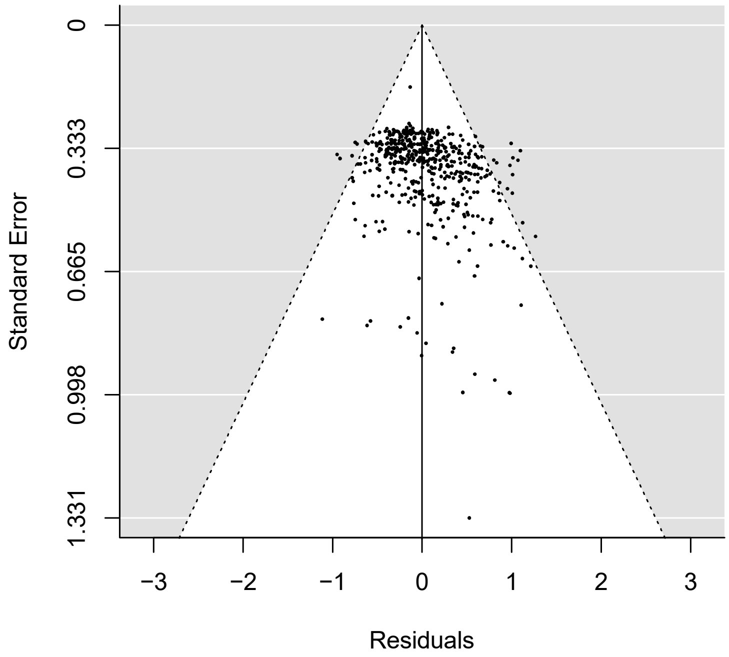

Figure S2. Funnel plot of the transformed effect sizes after the removal of outliers.

Supplementary Material S9

Deviation Contrasts of the Transformed Effect Sizes with the Outliers Removed

Table S2. Results of all Deviation Contrasts of the Transformed Effect Sizes after the Removal of Outliers

Contrast	*Mean*	95% CI	Δ*g*	Δ*g* (*se*)	Δ*g* (*t*)	Δ*g* (*p*)	Δ*g* (*p'*)
Average Age	*(B)* -0.019	[-0.032, -0.005]		(*se*) 0.007	(*t*) -2.731	.007**	
**Treatment**							
DBT	0.811	[0.693, 0.928]	-0.016	0.047	-0.346	.365	1.000
DBTmin	0.968	[0.791, 1.144]	0.140	0.078	1.799	.036*	.254
ST	0.995	[0.819, 1.172]	0.168	0.080	2.099	.018*	.164
TFP	0.801	[0.624, 0.978]	-0.026	0.075	-0.348	.364	1.000
MBT	0.984	[0.807, 1.160]	0.157	0.080	1.964	.025*	.200
TAU	0.537	[0.418, 0.655]	-0.291	0.051	-5.752	<.001***	<.001***
PDT	0.910	[0.740, 1.080]	0.083	0.074	1.125	.131	.653
CTBE	0.591	[0.355, 0.827]	-0.236	0.103	-2.286	.011*	.113
Spec. Other	0.833	[0.696, 0.970]	0.006	0.061	0.092	.463	1.000
CBT	0.998	[0.774, 1.222]	0.171	0.105	1.624	.052	.315
Th. Com	0.672	[0.114, 1.230]	-0.155	0.264	-0.588	.278	1.000
Grand Mean	*0.827*						
**BPD Domain**							
General severity	1.236	[1.112, 1.360]	0.409	0.049	8.272	<.001***	<.001***
Abandonment	0.745	[0.521, 0.968]	-0.083	0.093	-0.885	.188	.416
Interpersonal	0.784	[0.593, 0.975]	-0.043	0.078	-0.554	.290	.416
Identity Disturbance	1.012	[0.800, 1.224]	0.185	0.088	2.098	.018*	.073
Impulsivity	0.624	[0.486, 0.761]	-0.203	0.053	-3.862	<.001***	<.001***
Suicidality/Self-injury	0.623	[0.529, 0.717]	-0.204	0.039	-5.242	<.001***	<.001***
Affective Instability	1.097	[0.930, 1.265]	0.270	0.067	4.050	<.001***	<.001***
Emptiness	0.926	[0.708, 1.144]	0.099	0.091	1.088	.139	.416
Anger	0.627	[0.507, 0.747]	-0.200	0.044	-4.531	<.001***	<.001***
Dissociation	0.599	[0.434, 0.763]	-0.228	0.066	3.484	<.001***	.001**
Grand Mean	*0.827*						
**Outcome Type**							
Continuous	0.681	[0.594, 0.767]	-0.147	0.041	-3.562	<.001***	<.001***
Dichotomous	0.974	[0.811, 1.137]	0.147	0.041	3.562	<.001***	<.001***
Grand Mean	*0.827*						

Notes. * $p < .05$. ** $p < .01$. *** $p < .001$.

Supplementary Material S10

Chi-Square Test of the Predictors and Deviation Contrasts of the Transformed Effect Sizes for the Priority to Total Scale Sensitivity Analysis

Table S3. Chi Tests of all the Predictors in the Full and Final Models of the Transformed Effect Sizes for the Total Scale Sensitivity Analysis

**Variable**	$\mathcal{X}^2$	*df*	*Loglikelihood*	*p*
**Full Model**		***92***	***-191.780***	
Treatment	119.750	34	-251.655	<.001***
BPD Domain	230.128	35	-306.844	<.001***
Treatment * BPD domain	86.670	44	-235.115	.001**
Setting	2.271	90	-192.915	.321
Format	1.730	90	-192.645	.421
Quality	0.085	91	-191.822	.771
Trial type	1.174	90	-192.467	.503
Publication year	0.168	91	-191.864	.682
Country of testing	0.273	90	-191.916	.873
Male proportion	0.411	91	-192.985	.522
Analysis type	18.151	89	-200.856	<.001***
Mean age	11.518	91	-197.539	.001**
Medication policy	1.615	91	-192.588	.204
Substance use exclusion	2.927	90	-193.243	.232
Assessment type	3.209	89	-193.384	.361
Outcome type	7.245	91	-195.402	.007**
**Final Model**		***24***	***-248.487***	
Treatment	70.353	14	-283.664	<.001***
BPD Domain	145.335	15	-321.155	<.001***
Outcome type	10.982	23	-253.978	.002**
Mean age	7.640	23	-252.307	.006**

Note. * $p < .05$. ** $p < .01$. *** $p < .001$.

Table S4. Results of all Deviation Contrasts of the Transformed Effect Sizes for the Priority to Total Scale Sensitivity Analysis

Contrast	*Mean* (*g*)	95% CI	Δ*g*	Δ*g* (*se*)	Δ*g* (*t*)	Δ*g* (*p*)	Δ*g* (*p'*)
Average Age	(*B*)			(*se*)	(*t*)	(*p*)	
	-0.026	[-0.046, -0.007]		0.001	-2.703	.007**	
**Treatment**							
DBT	0.854	[0.695, 1.013]	-0.021	0.060	-0.351	.363	1.000
DBTmin	1.060	[0.831, 1.288]	0.184	0.099	1.864	.031*	.283
ST	1.273	[0.976, 1.569]	0.398	0.138	2.881	.002**	.021*

TFP	0.896	[0.649, 1.143]	0.021	0.102	0.204	.419	1.000
MBT	1.002	[0.772, 1.232]	0.127	0.101	1.252	.106	.739
TAU	0.545	[0.386, 0.705]	-0.330	0.062	-5.297	<.001***	<.001***
PDT	0.909	[0.700, 1.118]	0.034	0.086	0.397	.346	1.000
CTBE	0.670	[0.393, 0.947]	-0.205	0.117	-1.752	.040*	.322
Spec. Other	0.851	[0.668, 1.034]	-0.025	0.076	-0.322	.374	1.000
CBT	0.962	[0.671, 1.254]	0.087	0.133	0.657	.256	1.000
Th. Com	0.605	[-0.189, 1.399]	-0.270	0.372	-0.725	.234	1.000
Grand Mean	*0.875*						
**BPD Domain**							
General severity	1.337	[1.189, 1.485]	0.461	0.061	7.587	<.001***	<.001***
Abandonment	0.837	[0.485, 1.189]	-0.038	0.149	-0.256	.399	.556
Interpersonal	0.772	[0.426, 1.118]	-0.103	0.146	-0.706	.240	.556
Identity Disturbance	1.060	[0.726, 1.394]	0.185	0.141	1.310	.095	.382
Impulsivity	0.575	[0.391 ,0.760]	-0.300	0.074	-4.052	<.001***	<.001***
Suicidality/Self-injury	0.716	[0.588, 0.843]	-0.160	0.054	-2.981	.002**	.011*
Affective Instability	1.119	[0.873, 1.366]	0.244	0.101	2.424	.008**	.047*
Emptiness	1.010	[0.654, 1.367]	0.135	0.151	0.896	.185	.556
Anger	0.648	[0.494, 0.803]	-0.227	0.056	-4.020	<.001***	<.001***
Dissociation	0.677	[0.469, 0.886]	-0.198	0.085	-2.342	.010*	.049*
Grand Mean	*0.875*						
**Outcome Type**							
Continuous	0.723	[0.584, 0.862]	-0.152	0.047	-3.232	.001**	.001**
Dichotomous	1.027	[0.821, 1.234]	0.152	0.047	3.232	.001**	.001**
Grand Mean	*0.875*						

Notes. * $p < .05$. ** $p < .01$. *** $p < .001$.

Supplementary Material S11

Graphs of the Transformed Effect Sizes and the Priority to Total Scale Sensitivity Analysis

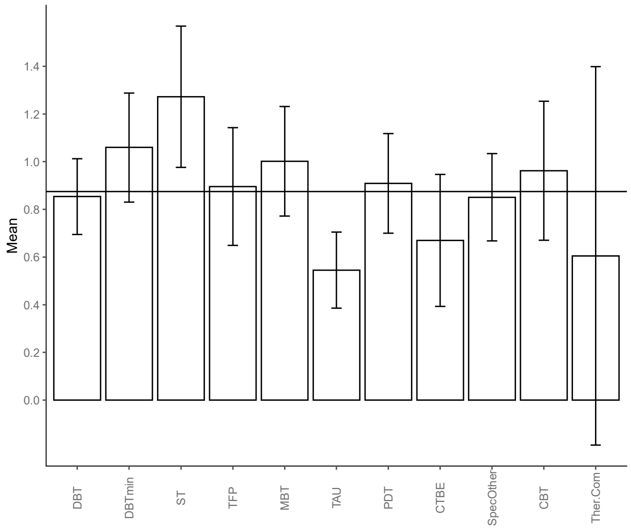

Figure S3. Bar graph of the estimated marginal means of the transformed effect sizes based on the log of time with 95% CIs estimated at one year of treatment for all treatments and the sensitivity analysis on the total scale results. The grand mean is depicted as a horizontal line at g = 0.875.

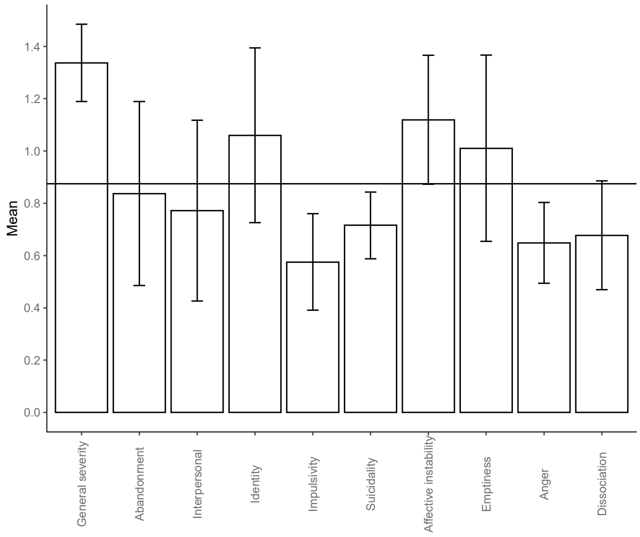

Figure S4. Bar graph of the estimated marginal means of the transformed effect sizes based on the log of time with 95% CIs estimated at one year of treatment for all BPD outcome domains and the sensitivity analysis on the total scale results. The grand mean is depicted as a horizontal line at g = 0.875.

Supplementary Material S12

Plots for the Final models of the Total Sensitivity Analysis on the Transformed Effect Sizes

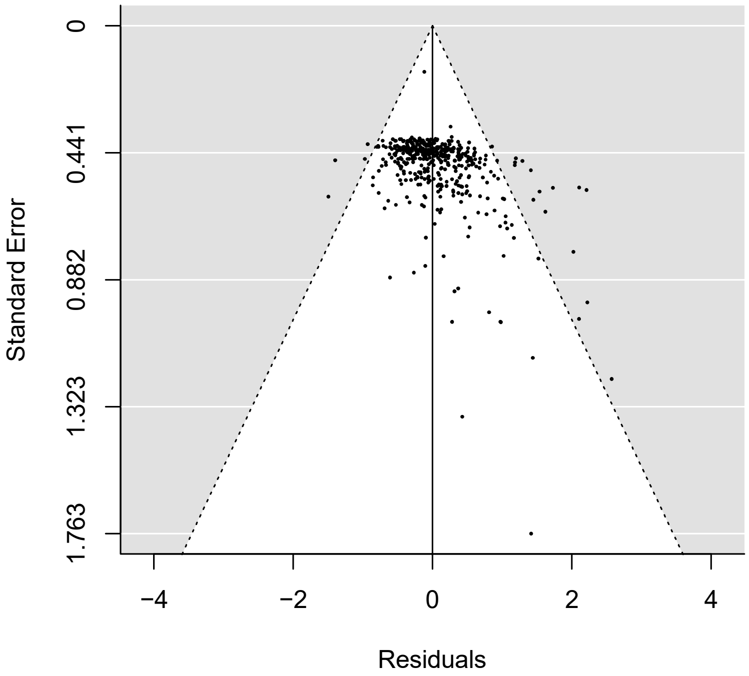

Figure S5. Funnel plot of the sensitivity analysis on the transformed effects sizes.

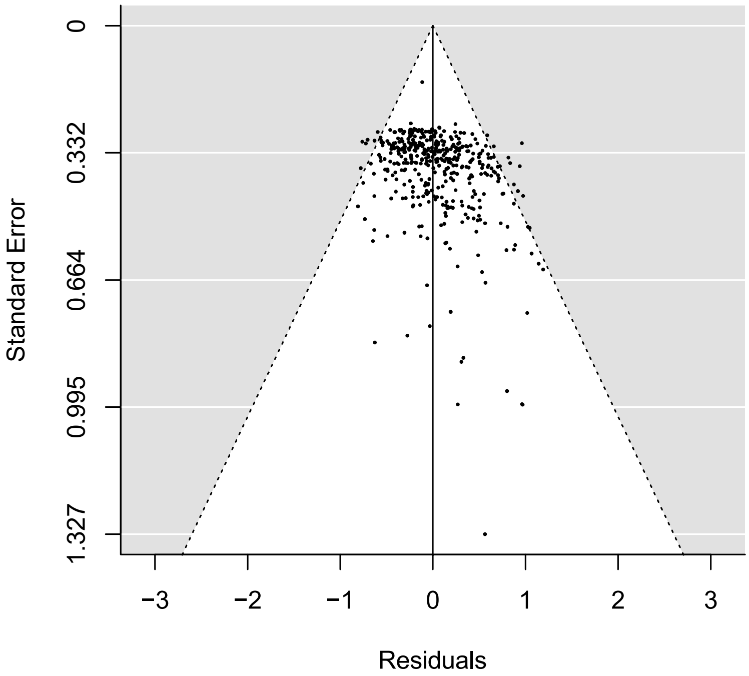

Figure S6. Funnel plot of the sensitivity analysis on the transformed effects sizes with the outliers removed.

Supplementary Material S13

Deviation Contrasts Transformed Effect Sizes Priority to Total Scale Sensitivity Analysis with the Outliers Removed

Table S5. Results of all Deviation Contrasts of the Transformed Effect Sizes for the Priority to Total Scale Sensitivity Analysis with Outliers Excluded

Contrast	*Mean*	95% CI	Δ*g*	Δ*g* (*se*)	Δ*g* (*t*)	Δ*g* (*p*)	Δ*g* (*p′*)
Average Age	*(B)*			(*se*)	(*t*)	(*p*)	
	-0.025	[-0.040, -0.011]		0.007	-3.483	.001**	
**Treatment**							
DBT	0.800	[0.668, 0.931]	-0.015	0.048	-0.321	.374	1.000
DBTmin	0.922	[0.730, 1.114]	0.107	0.082	1.310	.096	.573
ST	1.053	[0.808, 1.297]	0.238	0.111	2.142	.016*	.148
TFP	0.792	[0.586, 0.999]	-0.023	0.084	-0.272	.393	1.000
MBT	0.953	[0.770, 1.135]	0.138	0.078	1.758	.040*	.318
TAU	0.513	[0.382, 0.644]	-0.302	0.050	-5.986	<.001***	<.001***
PDT	0.885	[0.707, 1.062]	0.070	0.073	0.959	.169	.845
CTBE	0.577	[0.342, 0.812]	-0.238	0.099	-2.418	.008**	.080
Spec. Other	0.849	[0.699, 0.998]	0.034	0.062	0.541	.295	1.000
CBT	0.983	[0.747, 1.219]	0.168	0.107	1.570	.059	.410
Th. Com	0.640	[0.088, 1.192]	-0.175	0.260	-0.674	.250	1.000
Grand Mean	*0.815*						
**BPD Domain**							
General severity	1.254	[1.136, 1.372]	0.439	0.055	7.996	<.001***	<.001***
Abandonment	0.777	[0.452, 1.102]	-0.038	0.142	-0.268	.394	.496
Interpersonal	0.718	[0.399, 1.037]	-0.097	0.139	-0.697	.243	.496
Identity Disturbance	0.980	[0.676, 1.284]	0.165	0.133	1.240	.108	.432
Impulsivity	0.556	[0.402, 0.710]	-0.259	0.068	-3.792	<.001***	.001**
Suicidality/Self-injury	0.629	[0.533, 0.724]	-0.186	0.049	-3.803	<.001***	.001**
Affective Instability	1.050	[0.831, 1.269]	0.235	0.095	2.480	.007**	.041*
Emptiness	0.956	[0.626, 1.286]	0.141	0.144	0.974	.165	.496
Anger	0.603	[0.480, 0.727]	-0.212	0.053	-4.021	<.001***	<.001***
Dissociation	0.628	[0.448, 0.808]	-0.187	0.078	-2.400	.008**	.042*
Grand Mean	*0.815*						
**Outcome Type**							
Continuous	0.677	[0.571, 0.784]	-0.138	0.040	-3.424	<.001***	.001**
Dichotomous	*0.953*	[0.781, 1.125]	0.138	0.040	3.424	<.001***	.001**
Grand Mean	*0.815*						

Notes. * $p < .05$. ** $p < .01$. *** $p < .001$.

Supplementary Material S14

Sensitivity Analysis Repeating the Complete Main Analysis on Untransformed Effect Sizes

N.1.1 Main analysis

Model fit did not significantly improve when the number of Axis I and II disorders and ISCED level were added to the model, Dm(3, 153.63) = 0.07, p = .977, so we decided to exclude these variables and to continue with a complete dataset. Both the within-study variance (σ_u^2 = 0.123, $\mathcal{X}^2(1)$ = 557.83, p < .001), and the between-study variance (σ_u^2 = 0.132, $\mathcal{X}^2(1)$ = 82.02, p < .001), were significant. The percentage of sampling variance was 12.20%, the within-study variance was 42.25% and the between-study variance was 45.55%.

According to our model selection procedure, there were three equivalent models. The final model of the untransformed effect sizes consisted of the following predictors: 1) Treatment, 2) BPD outcome domain, 3) the log transformed treatment duration in weeks, 4) analysis type, 5) the mean age of the sample and 6) outcome type. Our other two models excluded either analysis type or mean age. The final model we chose was the model with the highest significant log-likelihood ratio test statistic. Table S6 shows the tests for the predictors in the full model and the final model. For our final model, the within-study variance (σ_u^2 = 0.076, $\mathcal{X}^2(1)$ = 248.414, p < .001, and the between-study variance (σ_u^2 = 0.039, $\mathcal{X}^2(1)$ = 21.620, p < .001, were significant. Sampling variance was 23.56%, within-study variance was 50.49% and between-study variance was 25.95%. Thus, the final model explained part of the heterogeneity between and within studies, although there was still considerable heterogeneity between and within studies.

Table S6. Chi-Square Tests of all the Predictors in the Full and Final Models of the Untransformed Effect Sizes

**Variable**	$\mathcal{X}^2$	*df*	*Loglikelihood*	*p*
**Full Model**		***107***	***-220.338***	
Weeks of Treatment	1.907	106	-223.335	.167
Treatment	165.598	35	-303.137	<.001***
BPD Domain	259.315	36	-349.995	<.001***
Treatment * BPD domain	122.987	45	-281.831	<.001***
Setting	2.347	105	-221.511	.309
Format	2.963	105	-221.819	.227
Quality	0.055	106	-220.366	.814
Trial type	0.802	105	-220.739	.670
Publication year	0.127	106	-220.401	.721
Country of testing	0.624	105	-220.650	.732
Male proportion	0.151	106	-220.414	.697
Analysis type	23.106	104	-231.891	<.001***
Mean age	12.855	106	-226.765	<.001***
Medication policy	0.105	106	-220.390	.746
Substance use exclusion	0.132	105	-220.404	.936
Assessment type	1.959	104	-221.317	.581

Outcome type	10.627	106	-225.651	.001**
**Final Model**		***28***	***-286.633***	
Weeks of Treatment	6.494	27	-289.878	.011*
Treatment	79.179	18	-326.222	< .001***
BPD Domain	140.511	19	-356.888	< .001***
Mean age	13.871	27	-293.568	<.001***
Analysis type	17.844	25	-295.555	<.001***
Outcome type	15.657	27	-204.456	<.001***

Note. * $p < .05$. ** $p < .01$. *** $p < .001$.

N.1.2 Deviation contrasts

The average grand mean was large (g = 0.926).

Treatment. All treatments appeared effective in reducing BPD symptomatology, with effect sizes ranging from medium to large. At one year, ST (g = 1.276), followed by MBT (g = 1.113), was related to treatment effects compared to the grand mean. TAU (g = 0.607) was less effective in reducing BPD symptoms compared to the average, but still had an average effect. All results are shown in Table S7 and the graph is shown in Figure S7.

BPD outcomes. At one year, the largest improvement compared to the average improvement over all domains was found for overall general severity (g = 1.309), followed by affective instability (g = 1.235). The least improvement was found for dissociation (g = 0.680), impulsive behaviors (g = 0.726), anger (g = 0.728) and suicidality/self-injury (g = 0.750). The estimated marginal means were medium to large; see Figure S8.

Analysis type. Compared to the average at 52 weeks of treatment, LOCF analysis (g = 0.633) was related to the smallest improvement in BPD outcomes, whereas ITT techniques from which the exact method was unclear (g = 1.230), was related to the highest effects. The estimated marginal means ranged from g = 0.633 to g = 1.230.

Outcome type. Continuous outcomes (g = 0.760) were related to smaller effects compared to the average, whereas dichotomous outcomes (g = 1.091) were related to larger effects. The estimated means were medium to large.

Table S7. Effects of Continuous Predictors and Results of all Deviation Contrasts of the Untransformed Effect Sizes at 1 Year

Contrast	*Mean (g)*	95% CI	Δ*g*	Δ*g* (*se*)	Δ*g* (*t*)	Δ*g* (*p*)	Δ*g* (*p'*)
Average Age	*(B)*			(*se*)	(*t*)	<.001***	
	-0.029	[-0.044, -0.013]		0.008	-3.548		
Log(Weeks)	*(B)*			(*se*)	(*t*)	.019*	
	0.105	[0.018, 0.192]		0.044	2.362		
**Treatment**							
DBT	0.886	[0.734, 1.037]	-0.040	0.056	-0.715	.238	.949

DBTmin	1.021	[0.824, 1.218]	0.096	0.085	1.130	.130	.777
ST	1.276	[1.064, 1.487]	0.350	0.092	3.813	<.001***	.001**
TFP	0.992	[0.775, 1.210]	0.067	0.089	0.748	.228	.949
MBT	1.113	[0.882, 1.344]	0.187	0.100	1.871	.031*	.279
TAU	0.607	[0.460, 0.755]	-0.318	0.058	-5.520	<.001***	<.001**
PDT	0.955	[0.759, 1.151]	0.029	0.083	0.353	.362	.949
CTBE	0.730	[0.449, 1.012]	-0.195	0.121	-1.615	.053	.427
Spec. Other	0.871	[0.703, 1.039]	-0.055	0.071	-0.768	.221	.949
CBT	1.090	[0.843, 1.337]	0.165	0.119	1.387	.083	.581
Th. Com	0.639	[-0.056, 1.334]	-0.286	0.325	-0.880	.190	.949
Grand Mean	*0.926*						
**BPD Domain**							
General severity	1.309	[1.153, 1.464]	0.383	0.051	7.450	<.001***	<.001***
Abandonment	0.830	[0.574, 1.086]	-0.096	0.102	-0.935	.175	.525
Interpersonal	0.962	[0.752, 1.171]	0.036	0.078	0.468	.320	.525
Identity Disturbance	1.044	[0.811, 1.277]	0.118	0.091	1.303	.097	.386
Impulsivity	0.726	[0.561, 0.891]	-0.199	0.056	-3.582	<.001***	.001**
Suicidality/Self-injury	0.750	[0.621, 0.879]	-0.175	0.041	-4.236	<.001***	<.001***
Affective Instability	1.235	[1.044, 1.425]	0.309	0.068	4.552	<.001***	<.001***
Emptiness	0.992	[0.748, 1.236]	0.066	0.096	0.692	.245	.525
Anger	0.728	[0.578, 0.877]	-0.198	0.046	-4.287	<.001***	<.001***
Dissociation	0.680	[0.491, 0.868]	-0.246	0.069	-3.537	<.001***	.001**
Grand Mean	*0.926*						
**Analysis Type**							
ITT Modern	0.908	[0.765, 1.051]	-0.017	0.059	-0.296	.384	.768
ITT LOCF	0.633	[0.456, 0.810]	-0.293	0.072	-4.088	<.001***	<.001***
ITT Unclear	1.230	[0.911, 1.548]	0.304	0.118	2.578	.005**	.015*
Completer	0.931	[0.796, 1.066]	0.006	0.056	0.102	.460	.768
Grand Mean	*0.926*						
**Outcome Type**							
Continuous	0.737	[0.642, 0.878]	-0.165	0.044	-3.748	.002**	.004**
Dichotomous	0.992	[0.898, 1.284]	0.165	0.044	3.748	.002**	.004**
Grand Mean	*0.864*						

Notes. * $p < .05$. ** $p < .01$. *** $p < .001$.

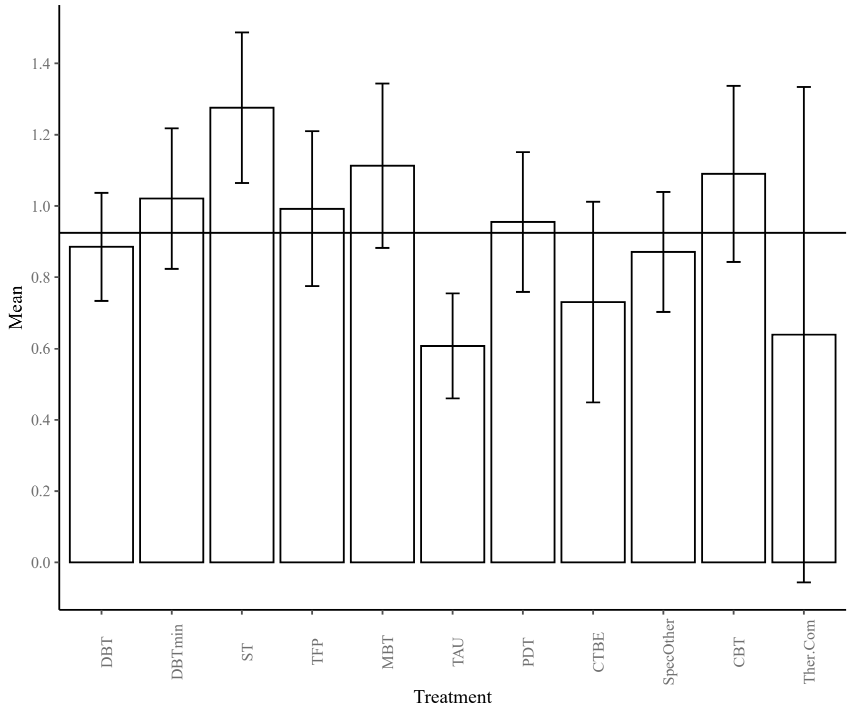

Figure S7. Bar graph of the estimated marginal means with 95% CI's at one year of treatment for all treatments for the untransformed effect sizes. The grand mean is depicted as a horizontal line at $g = 0.926$.

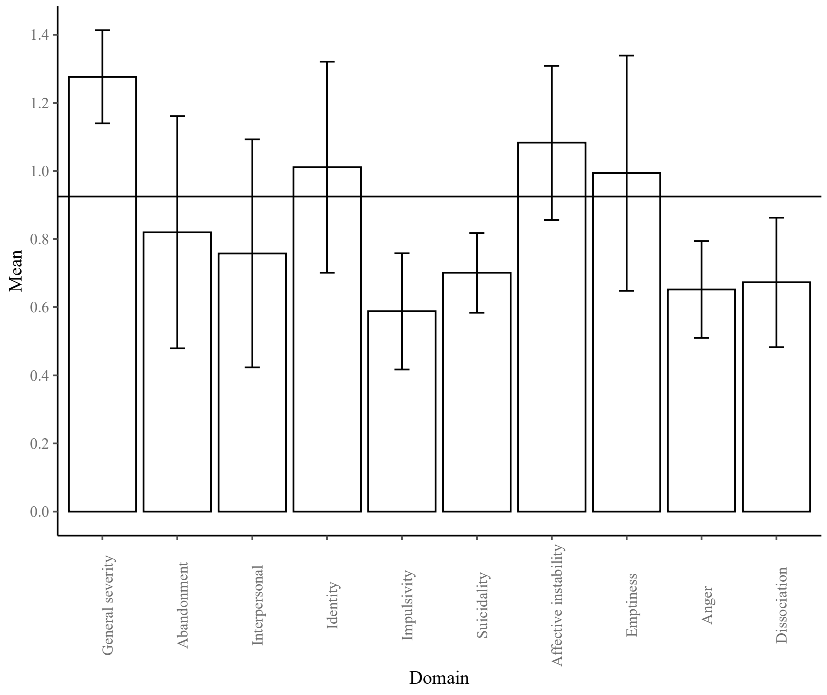

Figure S8. Bar graph of the estimated marginal means with 95% CI's at one year of treatment for all outcome domains for the untransformed effect sizes. The grand mean is depicted as a horizontal line at $g = 0.926$.

N.1.3 Treatment by domain interaction

We initially aimed to examine whether specific treatments are effective for particular BPD criteria, but this interaction was not included in our final model. We decided to explore these interactions (see Table S8), but we excluded treatments that did not contain at least

two effect sizes in a specific domain. At one year, TAU was associated with smaller effect sizes in improvement of general severity, impulsivity, suicidality, affective instability, anger and dissociation. ST was related to larger effect sizes for impulsivity, suicidality, anger, and dissociation. Lastly, compared to the average, DBT had smaller effect sizes for anger.

Table S8. Deviation Contrasts of the Interaction between Treatment and BPD Domain of the Untransformed Effect Sizes at 1 Year

Domain	*Mean* (*g*)	95% CI	Δ*g*	Δ*g* (*se*)	Δ*g* (*t*)	Δ*g* (*p*)	Δ*g* (*p'*)
**General Severity (*n* = *82*)**							
DBT	1.232	[0.673, 1.792]	-0.095	0.169	-0.563	.288	1.000
DBTmin	1.463	[0.821, 2.105]	0.135	0.241	0.560	.289	1.000
ST	1.279	[0.742, 1.816]	-0.049	0.178	-0.273	.393	1.000
MBT	1.458	[0.779, 2.137]	0.130	0.218	0.597	.276	1.000
TAU	0.646	[0.132, 1.160]	-0.682	0.141	-4.836	<.001***	.001**
PDT	1.644	[1.031, 2.257]	0.316	0.223	1.415	.081	.566
Spec. Other	1.293	[0.814, 1.771]	-0.035	0.193	-0.182	.428	1.000
CBT	1.607	[0.940, 2.275]	0.280	0.285	0.979	.165	.993
Grand Mean	*1.328*						
**Abandonment (*n* = *8*)**							
ST	0.768	[-8.147, 9.682]	0.212	0.586	0.362	.362	1.000
TAU	0.540	[-5.097, 6.176]	-0.016	0.405	-0.038	.488	1.000
Spec. Other	0.358	[-6.490, 7.207]	-0.197	0.529	-0.372	.387	1.000
Grand Mean	*0.555*						
**Interpersonal (*n* = *17*)**							
DBT	0.488	[-0.767, 1.743]	-0.461	0.513	-0.898	.199	.798
ST	1.703	[0.224, 3.182]	0.754	0.518	1.457	.094	.471
TAU	0.726	[-0.134, 1.587]	-0.222	0.374	-0.595	.285	.809
Spec. Other	0.626	[-0.465, 1.717]	-0.323	0.500	-0.646	.270	.809
CBT	1.200	[-1.418, 3.819]	0.251	0.907	0.277	.395	.809
Grand Mean	*0.949*						
**Identity (*n* = *14*)**							
ST	1.136	[-0.105, 2.377]	0.128	0.523	0.245	.408	1.000
TAU	0.420	[-0.839, 1.679]	-0.588	0.521	-1.127	.155	.622
Spec. Other	0.820	[-0.486, 2.126]	-0.187	0.591	-0.317	.382	1.000
CBT	1.654	[-2.781, 6.089]	0.646	1.478	0.437	.340	1.000
Grand Mean	*1.008*						
**Impulsivity (*n* = *50*)**							

DBT	0.547	[0.082, 1.012]	0.011	0.103	0.104	.459	1.000
DBTmin	0.553	[-0.028, 1.133]	0.016	0.191	0.085	.466	1.000
ST	0.899	[0.340, 1.457]	0.362	0.205	1.764	.043*	.302
TFP	0.534	[0.004, 1.064]	-0.002	0.153	-0.016	.494	1.000
TAU	0.234	[-0.202, 0.669]	-0.303	0.110	-2.749	.005**	.037*
PDT	0.555	[0.009, 1.102]	0.019	0.159	0.118	.453	1.000
Spec. Other	0.579	[0.050, 1.107]	0.042	0.145	0.289	.387	1.000
CBT	0.392	[-0.315, 1.098]	-0.145	0.252	-0.576	.284	1.000
Grand Mean	*0.537*						
**Suicidality (*n* = *184*)**							
DBT	0.726	[0.554, 0.898]	-0.096	0.069	-1.404	.081	.568
DBTmin	0.798	[0.559, 1.036]	-0.025	0.109	-0.229	.409	.782
ST	1.447	[1.012, 1.882]	0.624	0.196	3.183	.001**	.009**
TFP	0.858	[0.549, 1.168]	0.036	0.129	0.277	.391	.782
MBT	1.032	[0.772, 1.291]	0.209	0.115	1.823	.035*	.316
TAU	0.559	[0.381, 0.737]	-0.264	0.078	-3.383	<.001***	.005**
PDT	0.638	[0.285, 0.990]	-0.185	0.164	-1.126	.131	.748
CTBE	0.670	[0.356, 0.984]	-0.153	0.132	-1.156	.125	.748
Spec. Other	0.721	[0.515, 0.926]	-0.102	0.090	-1.133	.129	.748
CBT	1.053	[0.754, 1.353]	0.231	0.139	1.663	.049*	.393
Th. Com	0.547	[-0.129, 1.223]	-0.275	0.315	-0.875	.191	.748
Grand Mean	*0.823*						
**Affective Instability (*n* = *27*)**							
DBT	0.738	[0.119, 1.357]	-0.362	0.268	-1.352	.098	.526
DBTmin	1.679	[0.783, 2.576]	0.579	0.407	1.423	.088	.526
ST	1.599	[0.620, 2.577]	0.499	0.359	1.389	.093	.526
TFP	0.892	[-0.142, 1.926]	-0.208	0.420	-0.495	.314	.942
TAU	0.650	[0.031, 1.270]	-0.450	0.251	-1.791	.047*	.327
Spec. Other	0.996	[0.327, 1.665]	-0.104	0.298	-0.348	.366	.942
CBT	1.146	[-0.587, 2.879]	0.046	0.715	0.064	.475	.942
Grand Mean	*1.100*						
**Emptiness (*n* = *9*)**							
ST	1.639	[-5.453, 8.730]	1.135	0.568	2.000	.148	.329
TAU	-0.202	[-4.083, 3.680]	-0.705	0.253	-2.791	.110	.329
Spec. Other	0.073	[-5.989, 6.135]	-0.430	0.422	-1.020	.247	.329
Grand Mean	*0.503*						
**Anger (*n* = *184*)**							
DBT	0.536	[0.178, 0.893]	-0.108	0.063	-1.701	.046*	.235

DBTmin	0.727	[0.328, 1.127]	0.084	0.112	0.748	.228	.457
ST	1.077	[0.562, 1.592]	0.433	0.185	2.344	.011*	.075
TFP	0.817	[0.383, 1.251]	0.174	0.126	1.384	.085	.340
TAU	0.353	[-0.011, 0.717]	-0.291	0.079	-3.683	<.001***	.002**
PDT	0.525	[0.138, 0.911]	-0.119	0.095	-1.245	.108	.340
CTBE	0.397	[-0.050, 0.845]	-0.246	0.138	-1.781	.039*	.235
Spec. Other	0.716	[0.375, 1.057]	0.072	0.133	0.545	.294	.457
Grand Mean	*0.644*						
**Dissociation (*n* = 23)**							
DBT	0.345	[0.138, 0.552]	-0.068	0.079	-0.861	.202	.202
ST	0.926	[0.462, 1.390]	0.514	0.169	3.040	.004**	.018*
TAU	0.118	[-0.148, 0.385]	-0.294	0.111	-2.657	.009**	.028*
Spec. Other	0.261	[0.000, 0.522]	-0.151	0.105	-1.441	.086	.172
Grand Mean	*0.413*						

Notes. * $p < .05$. ** $p < .01$. *** $p < .001$.

N.1.4 Outliers and bias

The final model identified 30 outliers based on the studentized values, which is near the expected 5% (5.61%). Most of these outliers had effect sizes of $g > 1.3$, although two effect sizes were small ($g = -0.037$, $g = 0.277$). Nineteen outliers came from RCT designs and most of them were based on continuous outcomes ($n = 26$). Important to note is that the ST studies with outliers made up most of the ST studies. Several of these studies or treatment arms contained only one outlying effect size, while multiple effect sizes were included. However, three treatment arms contained either one or more effect sizes that were all identified as outliers (Jacob et al., 2018; Nordahl et al., 2005; Reiss et al., 2014-1). One study (Reiss et al., 2014-1) reported very large effect sizes ($g \geq 2.36$), while there were two studies of which all effect sizes were identified as outliers (Jacob et al., 2018 & Nordahl et al., 2005). The outliers from these last two studies had relatively small effect sizes (0.28 and 0.64 respectively). Taken together, these outcomes might have inflated or otherwise influenced the estimated effect sizes for the ST category, although not all effect sizes were very large.

Egger's test was significant, indicating a relationship between stronger effects and lower precision, $F(1, 508) = 376.437$, $p < .001$, $B = 5.895$. The funnel plot (Supplementary Material S15) showed a similar pattern as compared to the analysis of the transformed effect sizes. The trim-and-fill procedure suggested that there were 51 residuals missing on the right side of the funnel plot, whereas there were no effect sizes missing on the left side of the funnel plot. This did not seem to correspond to Egger's test, although the indices of Egger's test and trim-and-fill procedures do not always indicate similar conclusions [33]. Simulating these additional effect sizes would lead to an increase of the average effect size of $g = 0.077$.

After we removed all outliers, Egger's test was still significant, $F(1, 478) = 133.295$, $p < .001$, $B = 3.270$. The funnel plot of the final model without outliers is shown in Supplementary Material S15. All tests for the predictors in the final model were significant. For each predictor, the grand mean was 0.831. Overall, effect sizes for treatments decreased,

except for Therapeutic Community. In addition to ST and MBT, CBT was related to larger effect sizes compared to the grand mean. Also, CTBE was, in addition to TAU, related to smaller effect. For BPD outcome domains, the pattern of results was similar, and no differences were observed. The same applied to analysis type and outcome type (see Supplementary Material S16).

Supplementary Material S15

Funnel plots for the final models of the untransformed effect sizes

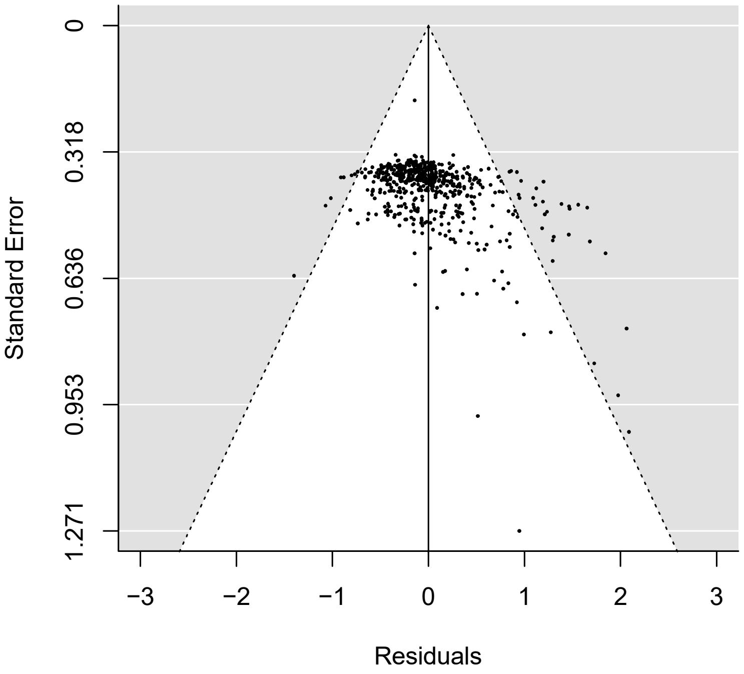

Figure S9. Funnel plot of the untransformed effect sizes.

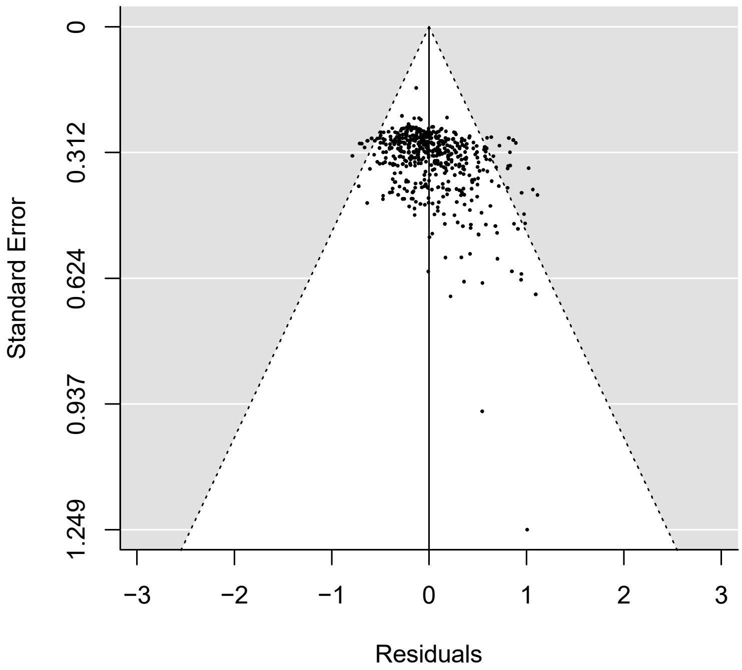

Figure S10. Funnel plot of the untransformed effect sizes with the outliers removed.

Supplementary Material S16

Deviation Contrasts Untransformed Effect Sizes with the Outliers Removed

Table S9. Effects of Continuous Predictors and Results of all Deviation Contrasts of the Untransformed Effect Sizes with Outliers Removed

Contrast	*Mean (g)*	95% CI	Δ*g*	Δ*g* (*se*)	Δ*g* (*t*)	Δ*g* (*p*)	Δ*g* (*p′*)
Average Age	*(B)*			(*se*)	(*t*)	<.001***	
	-0.024	[-0.036, -0.013]		0.006	-4.131		
Log(Weeks)	*(B)*			(*se*)	(*t*)	.006**	
	0.085	[0.025, 0.115]		0.044	2.362		
**Treatment**							
DBT	0.791	[0.679, 0.903]	-0.040	0.043	-0.933	.176	1.000
DBTmin	0.889	[0.738, 1.040]	0.058	0.066	0.875	.191	1.000
ST	1.114	[0.944, 1.284]	0.283	0.075	3.789	<.001***	.001**
TFP	0.850	[0.679, 1.021]	0.019	0.071	0.264	.396	1.000
MBT	1.001	[0.829, 1.173]	0.170	0.075	2.268	.012*	.090
TAU	0.545	[0.437, 0.654]	-0.286	0.045	-6.319	<.001***	<.001**
PDT	0.821	[0.667, 0.976]	-0.010	0.069	-0.143	.443	1.000
CTBE	0.614	[0.401, 0.827]	-0.217	0.092	-2.366	.009**	.083
Spec. Other	0.798	[0.673, 0.922]	-0.034	0.055	-0.612	.270	1.000
CBT	1.036	[0.850, 1.221]	0.204	0.089	2.288	.011*	.090
Th. Com	0.684	[0.153, 1.215]	-0.147	0.249	-0.591	.277	1.000
Grand Mean	*0.831*						
**BPD Domain**							
General severity	1.203	[1.082, 1.324]	0.372	0.044	8.505	<.001***	<.001***
Abandonment	0.792	[0.580, 1.003]	-0.040	0.089	-0.446	.328	.755
Interpersonal	0.908	[0.735, 1.081]	0.077	0.069	1.110	.134	.755
Identity Disturbance	0.818	[0.617, 1.020]	-0.013	0.084	-0.155	.438	.755
Impulsivity	0.658	[0.529, 0.786]	-0.174	0.047	-3.666	.001**	.001**
Suicidality/Self-injury	0.674	[0.579, 0.768]	-0.158	0.034	-4.572	<.001***	<.001***
Affective Instability	1.080	[0.926, 1.234]	0.249	0.060	4.149	<.001***	<.001***
Emptiness	0.889	[0.684, 1.094]	0.057	0.086	0.670	.252	.755
Anger	0.660	[0.547, 0.773]	-0.171	0.039	-4.347	<.001***	<.001***
Dissociation	0.631	[0.482, 0.781]	-0.200	0.059	-3.409	<.001***	.002**
Grand Mean	*0.831*						
**Analysis Type**							
ITT Modern	0.855	[0.748, 0.962]	0.024	0.037	0.641	.261	.261
ITT LOCF	0.586	[0.463, 0.710]	-0.245	0.049	-5.035	<.001***	<.001***

ITT Unclear	1.097	[0.889, 1.305]	0.266	0.075	3.532	<.001***	.001**
Completer	0.787	[0.683, 0.89]	-0.045	0.037	-1.222	.111	.222
Grand Mean	*0.831*						
**Outcome Type**							
Continuous	0.682	[0.600, 0.764]	-0.149	0.036	-4.184	<.001***	<.001***
Dichotomous	0.980	[0.830, 1.130]	0.149	0.036	4.184	<.001***	<.001***
Grand Mean	*0.831*						

Notes. * $p < .05$. ** $p < .01$. *** $p < .001$.

N.2 Sensitivity analysis – untransformed effect sizes total scale results

N.2.1 Sensitivity analysis main model

This sensitivity analysis was conducted using the untransformed effect sizes on total scale results if included studies reported these in addition to subscale results. Model fit did not improve when we added the three imputed variables with missing data, (Dm(3, 184.63) = 0.12, p = .949). Both the within-study variance (σ_u^2 = 0.100, $\mathcal{X}^2(1)$ = 318.783, p < .001) and the between-study variance (σ_u^2 = 0.177, $\mathcal{X}^2(1)$ = 100.577, p < .001) were significant. The percentage of sampling variance was 12.49%, the within-study variance was 31.56% and the between-study variance was 55.95%. Table S10 in Supplementary Material S17, upper part, shows the results for the full model.

For the untransformed effect sizes, the final model was almost equal to our first analysis. Apart from analysis type, all predictors were included. Our final model consisted of treatment, BPD domain, outcome type, mean age and the log transformed number of weeks. The chi-square tests for the final model are shown in Table S10, lower part. For the transformed effect sizes, the within-study variance (σ_u^2 = 0.047, $\mathcal{X}^2(1)$ = 97.350, p < .001), and the between-study variance (σ_u^2 = 0.078, $\mathcal{X}^2(1)$ = 60.360, p < .001) of the final model were also significant. The percentage of sampling variance was 24.00%, the within-study variance was 28.61% and the between-study variance was 47.39%.

N.2.2 Sensitivity analysis deviation contrasts

The average mean effect size was large; g = 0.855.

Treatment. At one year, ST (g = 1.220), followed by MBT (g = 1.033), was related to larger treatment effects compared to the grand mean. TAU (g = 0.549), followed by CTBE (g = 0.646), were related to smaller effects. All results are shown in Table S11. All estimated means were moderate to large. The graph can be found in Supplementary Material S18.

BPD outcomes. At one year, the highest improvement was found for general severity (g = 1.276), followed by affective instability (g = 1.083). The least improvement was found for impulsive behaviors (g = 0.588), followed by anger (g = 0.652), dissociation (g = 0.673) and suicidality (g = 0.701) (see Supplementary Material S17). The estimated marginal means ranged from medium to large.

Outcome type. Continuous outcomes (g = 0.707) were related to less improvement, compared to dichotomous outcomes (g = 1.004) at one year.

N.2.3 Sensitivity analysis outliers and bias

Egger's test was significant, $F(1, 420) = 166.537$, $p < .001$, $B = 3.963$. The trim-and-fill procedure indicated that 36 residuals were missing on the right side, whereas 0 were missing on the left side. This would increase the average effect size from 0.623 to 0.691. There were 24 outliers (5.4%). Thirteen outliers were from general severity, and 9 from suicidality. The funnel plots are shown in Supplementary Material S19.

After removing outliers, all predictors were significant according to the chi square tests. Egger's test was still significant, $F(1, 399) = 81.421$, $p < .001$, $B = 2.482$. The predictors had an estimated grand mean of 0.809. The deviation contrasts of treatment showed no differences compared to the analysis with outliers included, but the estimated means were generally smaller. The same applied to BPD domain and outcome type. Results and patterns were similar (see Supplementary Material S20).

Supplementary Material S17

Chi-Square Test of the Predictors and Deviation Contrasts of the Untransformed Effect Sizes for the Priority to Total Scale Sensitivity Analysis

Table S10. Chi-Square Tests of all the Predictors in the Full and Final Models of the Untransformed Effect Sizes

**Variable**	$\mathcal{X}^2$	*df*	*Loglikelihood*	*p*
**Full Model**		***93***	***-152.460***	
Weeks of Treatment	2.937	92	-153.929	.087
Treatment	124.120	35	-214.120	<.001***
BPD Domain	229.782	36	-267.351	<.001***
Treatment * BPD domain	88.518	45	-196.719	<.001***
Setting	3.062	91	-153.991	.216
Format	1.859	91	-153.390	.395
Quality	0.001	92	-152.461	.974
Trial type	1.467	91	-153.194	.480
Publication year	0.770	92	-152.846	.380
Country of testing	0.346	91	-152.633	.841
Male proportion	0.921	92	-152.921	.337
Analysis type	19.357	90	-162.139	<.001***
Mean age	11.388	92	-158.154	.001**
Medication policy	0.836	92	-152.878	.361
Substance use exclusion	0.782	91	-152.851	.676
Assessment type	3.837	90	-154.379	.280
Outcome type	8.960	92	-156.940	.003**
**Final Model**		***25***	***-208.938***	
Weeks of Treatment	5.255	24	-211.565	.022*
Treatment	70.338	15	-244.107	<.001***
BPD Domain	144.299	16	-281.087	<.001***

Outcome type	8.822	24	-213.348	.003**
Mean age	12.394	24	-215.134	<.001***

Note. * $p < .05$. ** $p < .01$. *** $p < .001$.

Table S11. Effects of Continuous Predictors and Results of all Deviation Contrasts of the Untransformed Effect Sizes for the Priority to Total Scale Sensitivity Analysis

Contrast	*Mean (g)*	95% CI	Δ*g*	Δ*g* (*se*)	Δ*g* (*t*)	Δ*g* (*p*)	Δ*g* (*p′*)
Average Age	*(B)*			(*se*)	(*t*)	.003**	
	-0.025	[-0.042, -0.009]		0.009	-2.985		
Log(Weeks)	*(B)*			(*se*)	(*t*)	.020*	
	0.119	[0.019, 0.218]		0.051	2.334		
**Treatment**							
DBT	0.828	[0.680, 0.976]	-0.028	0.057	-0.486	.314	1.000
DBTmin	0.965	[0.758, 1.173]	0.110	0.090	1.222	.111	.778
ST	1.220	[0.951, 1.490]	0.365	0.126	2.900	.002**	.020*
TFP	0.876	[0.640, 1.111]	0.020	0.098	0.208	.418	1.000
MBT	1.033	[0.813, 1.252]	0.177	0.098	1.810	.035*	.284
TAU	0.549	[0.401, 0.697]	-0.306	0.059	-5.236	<.001***	<.001**
PDT	0.903	[0.706, 1.101]	0.048	0.082	0.583	.280	1.000
CTBE	0.646	[0.383, 0.909]	-0.209	0.112	-1.871	.031*	.279
Spec. Other	0.828	[0.658, 0.999]	-0.027	0.072	-0.377	.353	1.000
CBT	0.947	[0.680, 1.214]	0.091	0.122	0.750	.227	1.000
Th. Com	0.614	[-0.134, 1.363]	-0.241	0.351	-0.687	.246	1.000
Grand Mean	*0.855*						
**BPD Domain**							
General severity	1.276	[1.140, 1.413]	0.421	0.057	7.412	<.001***	<.001***
Abandonment	0.820	[0.479, 1.161]	-0.036	0.145	-0.245	.403	.524
Interpersonal	0.758	[0.423, 1.093]	-0.098	0.142	-0.686	.247	.524
Identity Disturbance	1.011	[0.701, 1.321]	0.156	0.132	1.181	.119	.476
Impulsivity	0.588	[0.417, 0.758]	-0.268	0.070	-3.845	<.001***	.001***
Suicidality/Self-injury	0.701	[0.584, 0.817]	-0.155	0.051	-3.057	.001**	.008**
Affective Instability	1.083	[0.856, 1.309]	0.227	0.094	2.426	.008**	.047*
Emptiness	0.994	[0.648, 1.339]	0.138	0.148	0.936	.175	.524
Anger	0.652	[0.510, 0.794]	-0.203	0.053	-3.848	<.001***	.001**
Dissociation	0.673	[0.482, 0.863]	-0.182	0.078	-2.336	.010*	.050
Grand Mean	*0.855*						
**Outcome Type**							
Continuous	0.707	[0.578, 0.835]	-0.149	0.044	-3.414	<.001***	.001**
Dichotomous	1.004	[0.813, 1.196]	0.149	0.044	3.414	<.001***	.001**

Grand Mean	*0.855*

Notes. * $p < .05$. ** $p < .01$. *** $p < .001$.

Supplementary Material S18

Graphs of the Untransformed Effect Sizes and the Priority to Total Scale Sensitivity Analysis

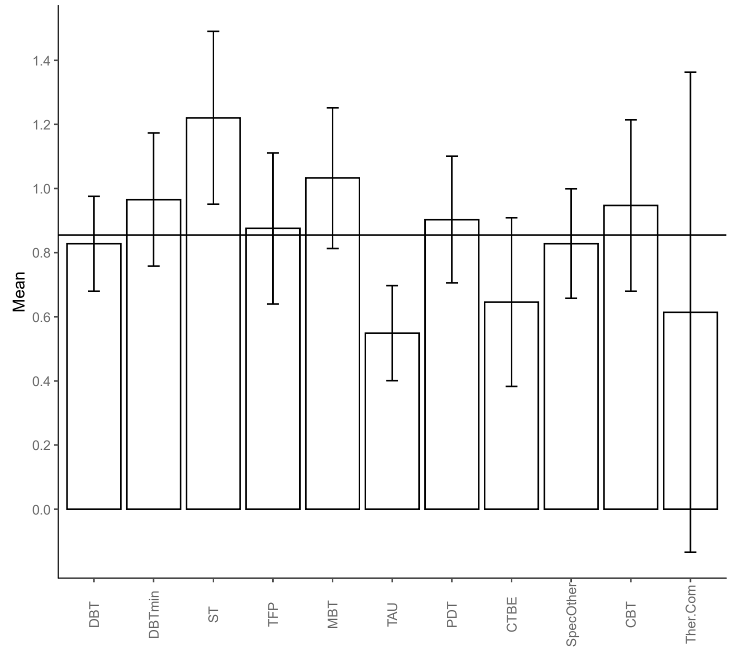

Figure S11. Bar graph of the estimated marginal means with 95% CI's at one year of treatment for all treatments for the untransformed effect sizes and the sensitivity analysis on the total scale results. The grand mean is depicted as a horizontal line at $g = 0.855$

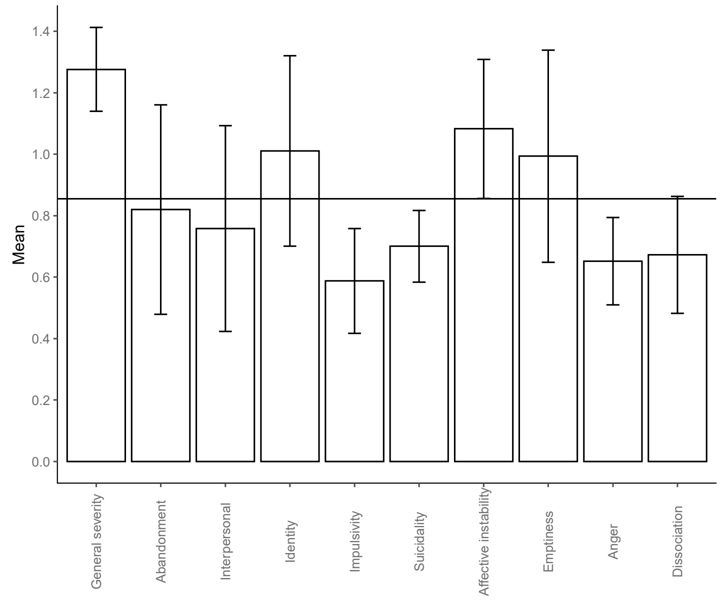

Figure S12. Bar graph of the estimated marginal means with 95% CI's at one year of treatment for all outcome domains for the untransformed effect sizes and the sensitivity analysis on the total scale results. The grand mean is depicted as a horizontal line at $g = 0.855$.

Supplementary Material S19

Funnel Plots for the Final models of the Total Sensitivity Analysis on the Untransformed Effect Sizes focused on Total Scale Results

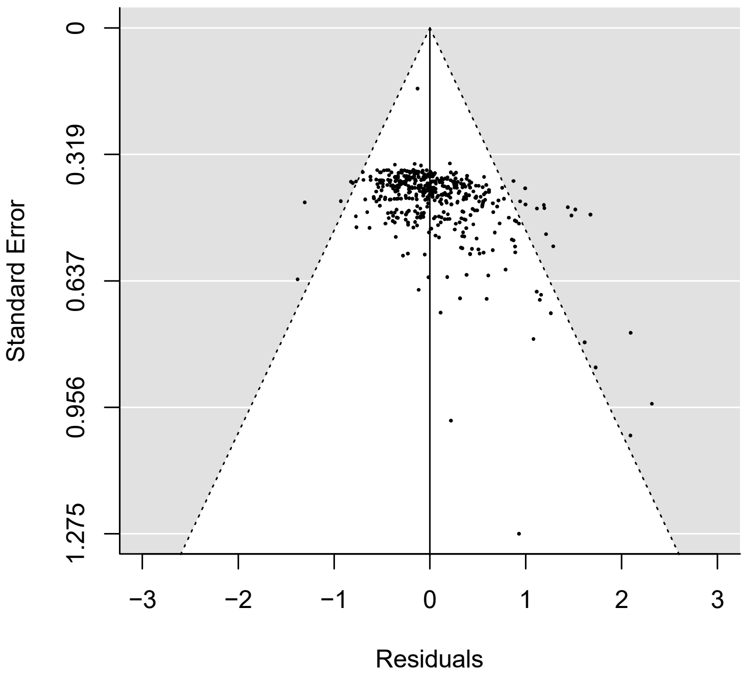

Figure S13. Funnel plot of the sensitivity analysis on the untransformed effects sizes focused on total scale results.

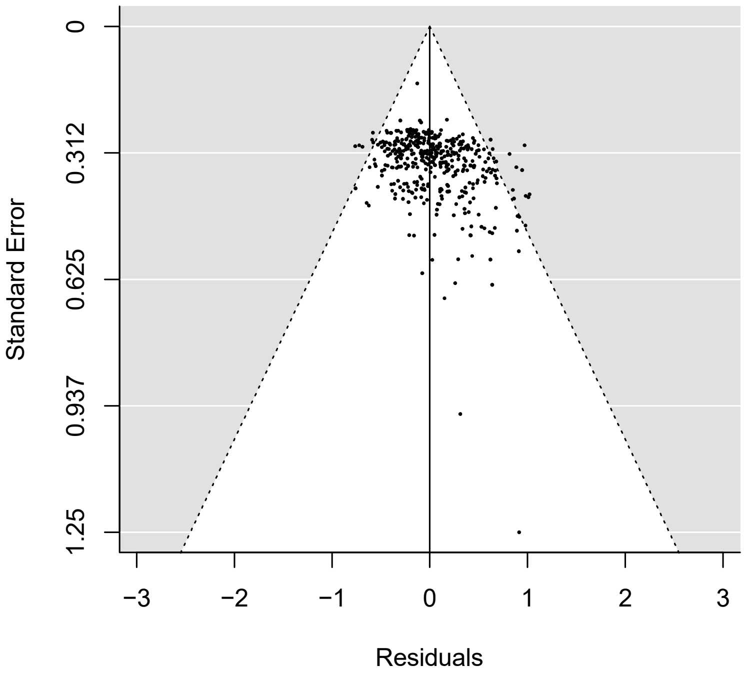

Figure S14. Funnel plot of the sensitivity analysis on the untransformed effect sizes focused on total scale results with outliers removed.

Supplementary Material S20

Deviation Contrasts Untransformed Effect Sizes Priority to Total Scale Sensitivity Analysis with the Outliers Removed

Table S12. Effects of Continuous Predictors and Results of all Deviation Contrasts of the Untransformed Effect Sizes for the Priority to Total Scale Sensitivity Analysis

Contrast	*Mean (g)*	95% CI	Δ*g*	Δ*g* (*se*)	Δ*g* (*t*)	Δ*g* (*p*)	Δ*g* (*p'*)
Average Age	*(B)*			(*se*)	(*t*)	.001**	
	-0.021	[-0.034, -0.008]		0.007	-3.226		
Log(Weeks)	*(B)*			(*se*)	(*t*)	.002**	
	0.121	[0.047, 0.196]		0.038	3.195		
**Treatment**							
DBT	0.783	[0.660, 0.907]	-0.026	0.046	-0.563	.287	1.000
DBTmin	0.867	[0.693, 1.041]	0.058	0.074	0.780	.218	1.000
ST	1.101	[0.866, 1.335]	0.292	0.106	2.755	.003**	.031*
TFP	0.786	[0.587, 0.985]	-0.023	0.082	-0.284	.388	1.000
MBT	0.988	[0.810, 1.167]	0.179	0.077	2.324	.010*	083
TAU	0.517	[0.395, 0.639]	-0.292	0.048	-6.069	<.001***	<.001**
PDT	0.851	[0.681, 1.021]	0.042	0.071	0.592	.277	1.000
CTBE	0.567	[0.344, 0.791]	-0.242	0.094	-2.566	.005**	.048*
Spec. Other	0.828	[0.689, 0.968]	0.019	0.059	0.323	.373	1.000
CBT	0.959	[0.747, 1.171]	0.150	0.096	1.557	.060	.421
Th. Com	0.652	[0.098, 1.206]	-0.157	0.260	-0.605	.273	1.000
Grand Mean	*0.809*						
**BPD Domain**							
General severity	1.194	[1.083, 1.306]	0.385	0.052	7.444	<.001***	<.001***
Abandonment	0.760	[0.444, 1.077]	-0.049	0.139	-0.352	.363	.725
Interpersonal	0.825	[0.491, 1.159]	0.016	0.147	0.106	.458	.725
Identity Disturbance	0.928	[0.648, 1.208]	0.119	0.123	0.968	.167	.667
Impulsivity	0.559	[0.415, 0.704]	-0.250	0.064	-3.880	<.001***	<.001***
Suicidality/Self-injury	0.628	[0.537, 0.718]	-0.182	0.047	-3.893	<.001***	<.001***

Affective Instability	1.018	[0.816, 1.220]	0.209	0.088	2.381	.009**	.044*
Emptiness	0.940	[0.619, 1.262]	0.131	0.141	0.929	.177	.667
Anger	0.610	[0.494, 0.725]	-0.200	0.049	-4.037	<.001***	<.001***
Dissociation	0.629	[0.465, 0.793]	-0.180	0.072	-2.509	.006**	.037*
Grand Mean	*0.809*						
**Outcome Type**							
Continuous	0.677	[0.576, 0.779]	-0.132	0.037	-3.533	<.001***	.001**
Dichotomous	0.941	[0.779, 1.103]	0.132	0.037	3.533	<.001***	.001**
Grand Mean	*0.809*						

Notes. * $p < .05$. ** $p < .01$. *** $p < .001$.
